# Mortality and Infectious Adverse Events in Neutropenic Patients Undergoing Gastrointestinal Endoscopic Procedures: A Systematic Review and Meta-Analysis

**DOI:** 10.1016/j.gastha.2026.100994

**Published:** 2026-05-08

**Authors:** Janak Bahirwani, Rishika Chugh, Ashley N. Tran, Amy Ogurick, Alyssa A. Grimshaw, Maria Ciarleglio, Yanhong Deng, Badr Al-Bawardy, Kenneth W. Hung, Loren Laine

**Affiliations:** 1 Department of Gastroenterology, Kadlec Regional Medical Center, Richland, Washington; 2 Department of Gastroenterology, Elson S. Floyd College of Medicine, Washington State University Tri-Cities Campus, Richland, Washington; 3 Department of Gastroenterology, University of California San Francisco, San Francisco, California; 4 Department of Gastroenterology, St Luke’s University Health Network, Bethlehem, Pennsylvania; 5 Division of Digestive Health, Yale School of Medicine, New Haven, Connecticut; 6 Yale Cushing/Whitney Medical Library Division, Harvey Cushing/John Hay Whitney Medical Library, Yale University, New Haven, Connecticut; 7 Division of Analytical Sciences, Yale School of Medicine, New Haven, Connecticut; 8 Division of Digestive Health, Yale School of Medicine, New Haven, Connecticut; 9 Division of Digestive Health, King Faisal Specialist Hospital, Riyadh, Saudi Arabia; 10 VA Connecticut Health Care System, West Haven, Connecticut

**Keywords:** Neutropenia, Endoscopy, Immunosuppression, Infection

## Abstract

**Background and Aims:**

The safety of gastrointestinal endoscopy in neutropenic patients remains unestablished. Gastrointestinal and infectious disease society guidelines indicate that infectious adverse events are increased after endoscopy in neutropenic patients. We performed a systematic review and meta-analysis to assess the safety of endoscopy in neutropenic patients.

**Methods:**

Cochrane Library, Embase, Google Scholar, MEDLINE, PubMed, Scopus, and Web of Science were searched through September 2025 for studies in neutropenic patients (absolute neutrophil count <1000 cells/μL) undergoing endoscopy with outcome data on infections, mortality, or fever. Conference abstracts from January 2017 to September 2025 were also searched. Two reviewers independently identified studies meeting inclusion criteria, performed data extraction, and assessed risk of bias. Coprimary outcomes were 30-day infection-related mortality and infectious adverse events within 7 days of endoscopy. Secondary outcomes were new bacteremia and new fever within 7 days of endoscopy. Random-effects meta-analyses were performed.

**Results:**

Six cohort studies met eligibility criteria (N = 1241 patients). Pooled incidence was 0.0% (95% confidence interval [CI] 0.00%–0.03%; I^2^ = 0.0%) for infection-related mortality and 7.3% (95% CI 0.00%–25.57%; I^2^ = 98.6%) for infectious adverse events, which consisted of bacteremia (2.4% [95% CI 0.03%–6.99%; I^2^ = 87.3%]) and new fever (4.8% [95% CI 0.00%–9.74%; I^2^ = 99%]). Preprocedural antibiotic use did not reduce infectious adverse events or bacteremia as compared to no antibiotic use: odds ratio = 2.97, 95% CI 0.47–18.67.

**Conclusion:**

Infection-related mortality within 30 days was 0% and infectious adverse events within 7 days occurred in 7.3% of neutropenic patients undergoing endoscopy. These findings suggest endoscopy maybe safely performed in appropriately selected neutropenic patients.

## Introduction

The safety of endoscopy in patients with neutropenia is unclear. The American Society for Gastrointestinal Endoscopy guidelines from 2015 state “Patients with severe neutropenia (absolute neutrophil count [ANC] <500 cells/μL) and advanced hematologic malignancies are at increased risk for bacteremia and sepsis after gastrointestinal (GI) endoscopy. The protective effect of prophylactic antibiotics in this patient population has not been well studied. However, this practice seems logical, especially in patients undergoing endoscopic procedures that are associated with a high risk of bacteremia.”^1^ The British Society of Gastroenterology guidelines indicate that “neutropenia predisposes to sepsis after endoscopy” and that afebrile patients with ANC <0.5 × 10^9^/L should be offered antibiotic prophylaxis for procedures known to be associated with a high risk of bacteremia.^2^ In contrast, the Infectious Diseases Society of America guidelines regarding neutropenic patients with cancer state that “diagnostic endoscopy rarely causes bacteremia.”^3^ Other studies state it is likely safe to proceed with endoscopy at varying levels of neutropenia.^3^^,^^4^

A systematic review from 2015 explored the safety of endoscopy in neutropenic cancer patients.^5^ They identified only 3 studies providing data on infectious adverse events; 2 examined only pediatric populations,^6^^,^^7^ while the third study examined only patients with leukemia and did not define the degree of neutropenia.^8^ Based on these very limited data, the authors suggested it is safe to perform endoscopy in neutropenic patients, although prophylactic antibiotic administration should be considered in some cases. Since the 2015 systematic review and the aforementioned guidelines from 2009 to 2015, additional studies have become available that should allow us to improve the precision of outcome estimates and potentially alter conclusions.

A recent systematic review by Loganathan et al^9^ identified 4 studies with neutropenic patients,^4^^,^^8^^,^^10^^,^^11^ and reported a pooled infectious adverse event rate of 6.8%. However, this review searched a limited number of databased and the degree of neutropenia was not specified for one of these studies. Since that publication, we identified additional studies to allow for more precise outcome estimates.

We therefore performed an updated, comprehensive systematic review including searches of additional databases to assess the safety of endoscopy in adult patients with neutropenia and the utility of antibiotic prophylaxis.

## Methods

This systematic review and meta-analysis was conducted according to the Preferred Reporting Items for Systematic Reviews and Meta-Analyses and Meta-analysis of Observational Studies in Epidemiology guidelines (Supplementary Appendix A).^12^^,^^13^ The study protocol was registered a priori on the International Prospective Register of Systematic Reviews (CRD42020155664). Institutional review board approval was not required for this meta-analysis because it analyzed only publicly available data from previously published studies and did not directly involve human participants.

### Data Sources and Search Strategy

Bibliographic databases (Cochrane Library, Google Scholar, Ovid Embase, Ovid MEDLINE, PubMed, Scopus, Web of Science Core Collection) were searched through September 9, 2025, for studies in neutropenic patients (ANC <1000 cells/μL) undergoing endoscopy. An extensive search strategy was developed by 2 authors, including a medical librarian and reviewed by a second librarian using Peer Review of Electronic Search Strategies.^14^ The search was restricted to English language citations, human subjects, and adults (Supplementary Appendix B: Search Strategies).

Titles and abstracts were independently reviewed by 3 reviewers (J.B., A.T., R.C.). Any citation considered potentially relevant underwent dual independent full-text review. Disagreements regarding study selection were resolved by consensus. Two authors (B.A., K.H.) served as final arbiters in the event of unresolved disagreement. Conference abstracts from Digestive Disease Week, American College of Gastroenterology Annual Scientific Meeting, and United European Gastroenterology Week from January 2017 to September 2025 also were searched by 3 reviewers (J.B., R.C., A.O.).

### Study Selection

Studies were limited to cohort studies (single arm or comparative) and randomized controlled trials. The population was limited to adult patients (≥18 years) with ANC less than 1000 cells/μL undergoing endoscopy. Because our outcomes are incidences, case-control studies, case series and case reports were excluded as these do not allow determination of incidences. Unpublished data were excluded with the exception of conference abstracts between January 2017 and September 2025.

### Data Extraction and Quality Assessment

Two reviewers independently (R.C./A.T., R.C./A.O., R.C./J.B., A.T./J.B.) extracted data and performed risk-of-bias assessment for each study using a standardized form. Disagreements were resolved by consensus. If consensus was not reached, a third independent reviewer (A.T., A.O., R.C.) served as final arbiter. Authors were not contacted for additional unpublished data. The Newcastle-Ottawa Scale for cohort studies was used to evaluate methodological quality of studies.^10^ This scale assigns a maximum of 9 stars for lowest risk of bias in the following 3 domains: selection of study groups (4 stars); comparability of study groups (2 stars); and ascertainment of outcomes (3 stars). Risk of bias was determined by comparing the total Newcastle-Ottawa Scale scores categorized into the following 3 groups: very high risk-of-bias-0-3; high risk-of-bias-4-6; and low risk-of-bias-7-9.^15^

### Study Outcomes

Primary outcomes included infection-related mortality within 30 days and infectious adverse events within 7 days following endoscopy. Secondary outcomes were new bacteremia and new-onset fever within 7 days of endoscopy. We also assessed the outcome of infectious adverse events in patients receiving and not receiving antibiotics prior to endoscopy. Characteristics of study populations included age, sex, race, history of malignancy (solid or hematologic), recent stem cell transplant, immunosuppressant use, and neutropenia. Immunosuppressants were defined as biologic therapies, chemotherapy, and steroids.

Procedural details including type of procedure (esophagogastroduodenoscopy [EGD], colonoscopy, sigmoidoscopy, enteroscopy, endoscopic retrograde cholangiopancreatography, endoscopic ultrasound, percutaneous endoscopic gastrostomy [PEG] tube placement) and procedural interventions (biopsy, hemostatic therapy, and sphincterotomy) were extracted. Data on use of antibiotics immediately prior to or periprocedure, when available, were recorded. Indications for procedures were separated into the following categories: anorexia/weight loss; nausea/vomiting; diarrhea; overt GI bleeding or anemia; dysphagia/odynophagia; abdominal pain; and other. Endoscopic and histologic findings were recorded as the following: ulcer; varices; other bleeding lesion; graft vs host disease; cytomegalovirus; other viral infection; candidiasis; other fungal infection; and *Clostridium difficile*.

Infection details were extracted, including the timing of infection in relation to the procedure and how each study defined an infection. We defined an infectious adverse event as new fever, new bacteremia, or PEG tube site infection following endoscopy with fever and bacteremia not present prior to endoscopy.

### Data Synthesis and Statistical Analysis

Random-effects model meta-analysis was employed using the inverse variance method. A random-effects model was chosen because marked heterogeneity across studies was anticipated given the likely great variability in populations, endoscopic procedures, and assessment of infections; a prior meta-analysis showed marked statistical heterogeneity for our primary outcome.^9^ Proportions were transformed using Freeman-Tukey double arcsine transformation for variance stabilization. Due to marked variation in sample size and several zero numerators in individual studies, a generalized linear mixed model analysis was also performed for sensitivity assessment; this yielded similar results to the Freeman-Tukey double arcsine transformation. However, particularly when evaluating new fever, the generalized linear mixed model analysis yielded an upper bound of zero for the confidence interval (CI). We therefore opted for the Freeman-Tukey double arcsine transformation.

The final pooled proportions and CIs were back-transformed for ease of interpretation. Heterogeneity between studies was assessed using the I^2^ statistic and the *Q test* with substantial heterogeneity defined as an I^2^ > 50% or *P* < .10, respectively. Publication bias was evaluated using visual inspection of a funnel plot for asymmetry. Forest plots were constructed to summarize pooled estimates and to graphically display results from individual studies.

## Results

**Figure 1:**
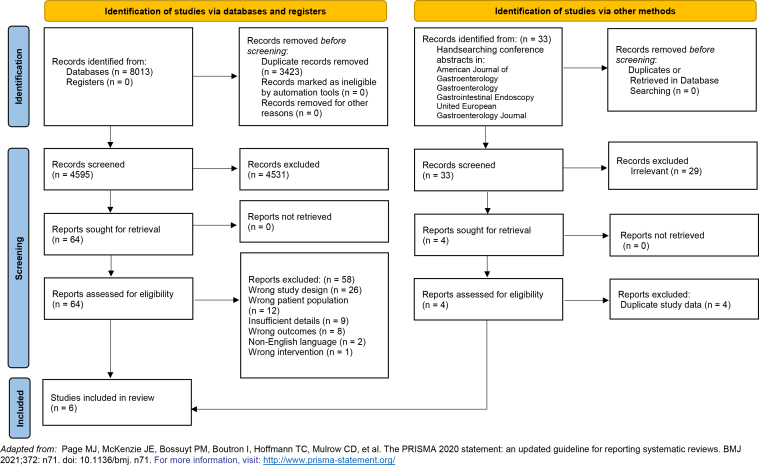
PRISMA flow diagram summarizing study selection. PRISMA, Preferred Reporting Items for Systematic Reviews and Meta-Analyses.

A total of 4595 unique citations were identified from the systematic search, and 4 citations were identified through hand search of conference abstracts. After title and abstract review, 64 studies were selected for full-text review. Studies that were reviewed in full text review but not included with specific reason for exclusion are provided in Supplementary Appendix C. A total of 6 studies met the inclusion criteria (Figure 1).^4^^,^^10^^,^^11^^,^^16^, ^17^, ^18^ No additional relevant studies were identified in our review of conference abstracts. All the 6 studies were single-arm cohorts. While Liu et al^17^ identified as a 2-armed cohort study (including patients with and without gastrointestinal graft vs host disease), for purposes of our study this was interpreted as a single-arm cohort as all patients included in our analysis had undergone hematopoietic stem cell transplant and were neutropenic. The 6 studies included 1241 procedures performed on neutropenic patients (range 10–675) (Table). The follow-up period for infectious adverse events related to endoscopy was 1–7 days with a median of 5 days. The follow-up period for mortality was 3–30 days with a median of 30 days.

**Table:** Study and Population Characteristics

Study	Abu-Sbeih, 2019	Isenberg, 2022	Kaw, 1993	Liu, 2013	Shin, 2022	Vishny, 1994
Location	United States	Israel	United States	United States	South Korea	United States
Study design	Cohort	Cohort	Cohort	Case-control	Cohort	Cohort
Total procedures performed on neutropenic patients	675	167	10	40	313	36
Mean age (y)	57.9	56.0	37.1	43.8	49.0	31.5
Male (%)	59	45.5	45	54	56	44
Duration follow-up (d)						
For infectious adverse events	7	2	–	7	7	3
For infection-related mortality	30	7	–	–	–	3
Malignancy diagnosis, n (%)	675 (100)	21 (12)	10 (100)	40 (100)	307 (98)	36 (100)
Solid	246 (36)	–	–	0 (0)	0 (0)	0 (0)
Hematologic	429 (64)	–	–	40 (100)	307 (100)	36 (100)
Stem cell transplant recipient, n (%)	147 (22)	34 (20)	10 (100)	40 (100)	74 (24)	36 (100)
Immunosuppressant recipient, n (%)	184 (27)	143 (73)	–	40 (100)	64 (20)	36 (100)
ANC at time of procedure, n (%)		<1000 but not subclassified	<1000 but not subclassified	<1000 but not subclassified		
500–1000 cells/μL	420 (62)	–	–	–	139 (44)	0 (0)
<500 cells/μL	255 (38)	–	–	–	174 (56)	36 (100)
Antibiotic use, n (%)	485 (72)	94 (56)	7 (70)	–	300 (96)	36 (100)

### Population Characteristics

Data on malignancy diagnosis were available in all the 6 studies; 88% (N = 1089) of procedures were performed in patients carrying a cancer diagnosis as follows: 812 with a hematologic malignancy and 246 with a solid malignancy.^4^^,^^10^^,^^11^^,^^16^, ^17^, ^18^ For 31 procedures, the type of malignancy (hematologic vs solid) was unknown. Five of the 6 studies evaluated the impact of immunosuppressant use; 37.9% (N = 467) of procedures in these studies were performed on patients receiving immunosuppressants.^4^^,^^10^^,^^11^^,^^17^^,^^18^ Data on history of stem cell transplant were available in all the 6 studies; 27.4% (N = 341) of procedures were performed on patients who were stem cell transplant recipients. All studies had patients with an ANC count <1000 cells/μL. Degree of neutropenia was further stratified in the following 3 studies or 1024 out of the 1241 total procedures across all the 6 studies: 54.5% (N = 559) with ANC 500–1000 cells/μL; 32.8% (N = 336) with ANC <500 cells/μL; and 12.7% (N = 129) with ANC <200 cells/μL.^4^^,^^11^^,^^18^ We excluded Kaw et al^16^ from fever analysis because it is not clear if the 1 patient who developed a fever after undergoing a flexible sigmoidoscopy 3 days posttransplant was neutropenic or not. As detailed in this paper, this specific patient’s thorough work-up for infectious source was negative, suggesting that the fever was not related to an infectious adverse event from endoscopy.

### Procedures and Interventions

The number of each procedure type was not consistently indicated. Four studies provided data on procedural intervention. Among these studies, 59% (N = 705) of procedures involved endoscopic intervention as follows: 8.6% (N = 46) included hemostatic therapy and 77.1% (N = 544) included biopsy. Sphincterotomy and PEG tube placement were also performed, although their frequencies were not specified. Antibiotic use prior to endoscopy was noted in 5 out of the 6 studies, with 76.9% (N = 923) of procedures in these studies done after antibiotic administration.^4^^,^^10^^,^^11^^,^^16^^,^^18^ Indications for procedures included nausea and/or vomiting, anemia, overt signs of luminal bleeding, dysphagia, odynophagia, and other. The frequencies of procedures done for each of these indications were mentioned in 2 studies.^4^^,^^11^

### Endoscopic and Histologic Findings

Endoscopic and histologic findings across all studies included peptic ulcer, varices, other bleeding lesions, graft vs host disease, cytomegalovirus, candidiasis, *C difficile*, nonspecific inflammation (including gastritis and esophagitis), and other. The frequency of these findings was not consistently described.

### Primary and Secondary Outcomes

**Figure 2:**
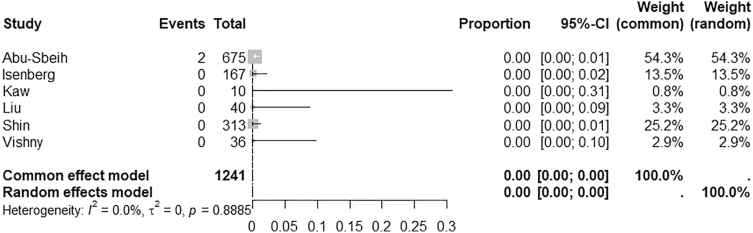
Forest plot of incidence of infection-related mortality.

**Figure 3:**
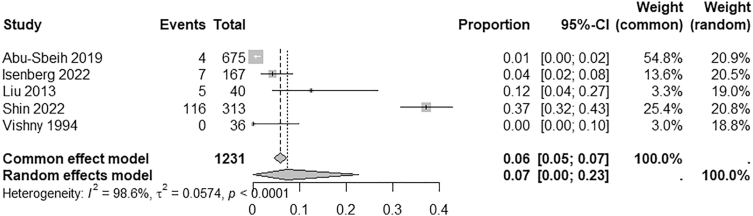
Forest plot of infectious adverse events.

**Figure 4:**
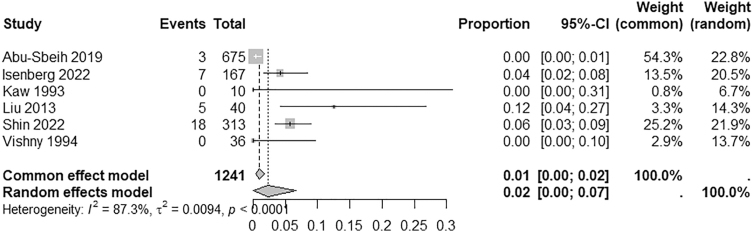
Forest plot of new bacteremia.

**Figure 5:**
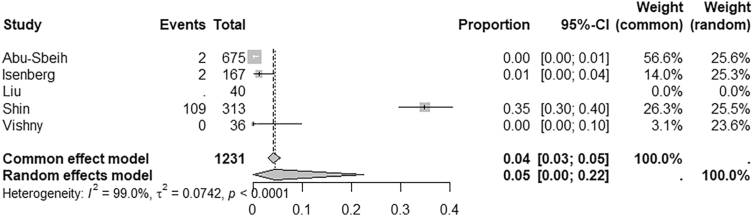
Forest plot of new fever.

Infection-related mortality within 30 days (duration of follow-up 3–30 days) was noted in 3 studies with pooled incidence of 0.0% (95% CI 0.0%–0.03%; I^2^ = 0.0%) (Table, Figure 2).^4^^,^^10^^,^^18^ Infectious adverse events within 7 days (duration of follow-up 2–7 days) were reported in 5 studies with pooled incidence of 7.3% (95% CI 0.00%–27.57%; I^2^ = 98.6%) (Table, Figure 3).^4^^,^^10^^,^^11^^,^^17^^,^^18^ Infectious adverse events include both documented new infection and new fever, and we also assessed these elements individually. New bacteremia was reported in all the 6 studies, and the pooled incidence was 2.4% (95% CI 0.03%–6.99%; I^2^ = 87.3%) (Figure 4). New fever was noted in 5 studies, and the pooled incidence was 4.8% (95% CI 0.00%–9.74%; I^2^ = 99%) (Figure 5).^4^^,^^10^^,^^11^^,^^17^^,^^18^

PEG tube placements were included in the study of Abu-Sbeih et al,^4^ with 2 of 42 patients undergoing this procedure developing PEG site infection. PEG tube placements were not explicitly mentioned as an endoscopic intervention in the remaining studies and therefore not measured separately as an outcome.

Data on antibiotic use prior to endoscopy related to outcomes of infectious adverse events or bacteremia were available in 5 studies.^4^^,^^10^^,^^11^^,^^16^^,^^18^ Kaw et al^16^ had a total of 10 bone marrow transplant recipients undergoing EGD, flexible sigmoidoscopy, or colonoscopy. Seven of these patients were on antibiotics at the time of their procedure; no patients, including those without antibiotics, developed bacteremia. Vishny et al^18^ included 36 bone marrow transplant recipients who were all receiving antibiotics and had an EGD, with no infectious adverse events noted. Abu-Sbeih et al^4^ had 485 procedures in patients receiving antibiotics and the following 4 of those patients developed infectious adverse events within 1 week: 2 of these were PEG tube site infections and 2 were fever of unknown origin (2 others developed gram-negative sepsis but had infections present at time of endoscopy). None of the 190 patients not receiving antibiotics in the study of Abu-Sbeih et al developed infectious adverse events within 1 week. Isenberg et al^10^ reported rates of bacteremia to be similar in those receiving preprocedural antibiotics (4/94 (4.3%) and those not receiving antibiotics 3/73 (4.1%). The study of Shin et al^11^ had 300 (96%) of 313 neutropenic patients who were receiving antibiotics at the time of the procedure; all infectious events occurred in those on antibiotics (116 [38.7%]). Most of these studies did not provide specific information on the indications for antibiotic use; only the study of Shin et al^11^ mentioned indication, stating that “most” patients received antibiotics for prevention of neutropenic fever and not for the prevention of endoscopy-related adverse events, while some received antibiotics to treat an underlying medical condition. Random-effects meta-analysis of the 4 studies comparing infectious adverse events or bacteremia in those receiving antibiotics vs those not receiving antibiotics revealed odds ratio = 2.97; 95% CI 0.47–18.67; and I^2^ = 45%.^4^^,^^10^^,^^11^^,^^16^

### Risk-of-Bias Assessment

All studies lacked a nonexposed cohort (neutropenic patients who did not undergo endoscopy) and therefore could only receive a maximum of 3 stars in the selection domain. (Supplementary Appendix D). For the selection domain, all studies received 3 stars. For the comparability domain, 2 studies received 1 star and 4 studies received 2 stars. For the outcome domain, 2 studies received 2 starts and 4 studies received 3 stars. Based on these findings, 5 out of the 6 studies were noted to have a low risk of bias (total score 7–9) and 1 study (Kaw et al) had a high risk of bias due to the total score being 6.

## Discussion

This systematic review and meta-analysis suggests that the risk associated with endoscopy in neutropenic patients is relatively low. The pooled incidence for one of our coprimary end points, infection-related mortality was 0.0% within 30 days. The pooled incidence for our other coprimary end point, infectious adverse events within 7 days, was 7.3%, but this end point included outcomes of both bacteremia, which had a pooled incidence of 2.4%, and fever, with pooled incidence of 4.8%.

An important limitation of all studies included in our systematic review is the lack of a control group of neutropenic patients not undergoing endoscopy. In neutropenic patients in general, the risk of infection is largely dependent on the duration and degree of neutropenia. In 1 study, the risk of infection was 14% in those with ANC between 500 and 1000 and 24%–60% in those with ANC <1000.^19^ Fever in neutropenic patients is estimated to occur in 10%–50% of patients with solid tumors and in >80% of those with hematologic malignancies.^20^ Eighty-eight percent of procedures in our review were performed on patients with malignancy, the majority being hematologic. Therefore, the infection risk related to endoscopy appears lower than the infection risk seen overall in neutropenic patients.

The meta-analysis for infection-related mortality shows no evidence of statistical heterogeneity (I^2^ = 0). In contrast, the meta-analysis of infectious adverse events shows dramatic heterogeneity with an I^2^ of 98.6%. In meta-analyses with marked heterogeneity, it is important to assess the potential causes of heterogeneity and consider whether the summary statistic from the pooled studies should be accepted. Visual examination of the forest plot for infectious adverse events (Figure 3) shows that the study of Shin et al is an outlier and the major driver for the increased number of infectious adverse events in our review. While new fever occurred in 35 of 109 patients, only a smaller proportion of these patients developed bacteremia (N = 11; 10%), suggesting the reported infectious adverse events were not clinically consequential in many patients. Shin et al reported poor performance status and myelodysplastic syndrome as risk factors for infectious adverse events on multivariable analysis in patients on antibiotics, and these characteristics were present in 37% and 57% of their patients, respectively. Further study is needed to determine if certain subsets of neutropenic patients have higher risk.

Guidelines from the British Society of Gastroenterology in 2009 state that neutropenia predisposes to sepsis after endoscopy endoscopy and recommend antibiotic prophylaxis for patients with severe neutropenia (ANC <500 cells/μL),^2^ and guidelines from the American Society for Gastrointestinal Endoscopy in 2015 state that patients with ANC <500 cells/μL and advanced hematologic malignancies are at increased risk for bacteremia and sepsis after GI endoscopy.^1^ Both guidelines cite only 1 article to support these statements, a study from 1990 in which 9 of 47 bone marrow transplant patients undergoing EGD developed clinically evident bacteremia.^21^ However, the population in this study was not patients with severe neutropenia—in fact, an inclusion criterion was ANC >500 cells/μL. Thus, these guideline statements regarding severe neutropenia were not based on data from patients with severe neutropenia. Since then, significantly more evidence has emerged. We found that 1155 (93%) of the cases included in our systematic review were from reports published after 2015, thereby enabling our review to provide far more evidence with more precise estimates of outcomes.

Societal guidelines suggest the use of prophylactic antibiotics when proceeding with endoscopy in patients with ANC <500 cells/μL again based on a single small retrospective study in patients who all had ANCs >500 cells/μL.^1^^,^^2^^,^^22^ In our meta-analysis, the use of antibiotic prophylaxis prior to endoscopy was stated in 5 of the 6 studies.^4^^,^^10^^,^^11^^,^^16^^,^^18^ Preprocedural antibiotic use was associated with a nonstatistically significant increase in infectious adverse events (odds ratio = 2.97; 95% CI 0.47–18.67), though this likely reflects confounding by indication, as antibiotics may have been preferentially administered to higher-risk patients. The observational nature of included studies and potential selection bias preclude definitive conclusions regarding antibiotic efficacy.

A 2007 abstract using the Nationwide Inpatient Sample database noted that among neutropenic patients with diagnostic indications such as GI bleeding, mortality was 49% less in those who underwent EGD and colonoscopy compared to those who did not.^23^ Appropriate use of endoscopy may therefore in fact decrease in-hospital and all-cause mortality. Studies in pediatric patients with neutropenia and immunocompromised states has yielded results similar to those seen in the adult population.^6^^,^^7^^,^^24^ In 1 study, both neutropenic and nonneutropenic pediatric patients underwent PEG tube placement with the same infection risk in both groups.^7^ In another group of 148 pediatric patients with ANC <1000, none developed infectious adverse events following endoscopy.^24^ And in a final study with 38 neutropenic pediatric cancer patients, there was only 1 infectious adverse event with fever and abdominal pain that resolved with 2 days of intravenous antibiotics.^6^

This study is not without limitations. Only 6 studies met inclusion criteria, demonstrating the limited data available and the need for additional large population-based studies. Out of these studies, 3 are clearly weighted more heavily than the others. The studies by Abu-Sbeih et al, Isenberg et al and Shin et al include 1155 procedures performed in neutropenic patients while the total number of procedures across the remaining 3 studies is only 86. Moreover, as mentioned above, no study had a comparator group. There is a known increased risk of infectious complications in neutropenic patients, regardless of whether or not they undergo endoscopy. Another limitation is the median short duration of follow-up for patients resulting in missed infectious adverse events. The follow-up period of infectious adverse events was only 1–7 days with a median follow-up of 5 days across all studies. It is conceivable that complications related to endoscopy may occur more than 1 week following endoscopy.

Further limitations include the lack of specific information on procedure type and procedural intervention. The available data did not allow us to assess if risk of infection varies with procedure type (eg, EGD, colonoscopy, PEG) or if infection increases with endoscopic intervention. In addition, immunosuppression is a known risk factor for infectious adverse events. While we know that 88% of patients included in our meta-analysis across all studies carried a diagnosis of malignancy and were neutropenic, other factors that may have led to further immunosuppression were not clear. The use of biologic or chemotherapeutic agents, steroids, or radiation therapy was not delineated. Finally, data were not available in the studies included in our systematic review to allow us to assess whether the risk varied with different severities or durations of neutropenia. Among the 1024 procedures were neutropenia was stratified, more than half had an ANC between 500 and 1000 cells/μL. This limits the generalizability of our findings to patients with severe neutropenia (ANC <500 cells/μL).

## Conclusion

This systematic review and meta-analysis demonstrates that the risk of endoscopic procedures in neutropenic adults may be lower than was suggested in the past and fails to document a benefit of preprocedural antibiotics. These findings highlight the need for large population-based studies on the safety of endoscopy in neutropenia, and further suggest that neutropenia should not be a barrier to endoscopy when it is otherwise indicated.

## Supplementary materials

Supplementary Appendix A–D

Extended PDF
