# Supplementary material for: Mortality and Infectious Adverse Events in Neutropenic Patients Undergoing Gastrointestinal Endoscopic Procedures: A Systematic Review and Meta-Analysis
Source: Gastro Hep Adv. 2026 May 8;5(8):100994. doi: 10.1016/j.gastha.2026.100994 (PMC13264052; doi:10.1016/j.gastha.2026.100994)
Supplement: Supplementary Appendix A–D [file mmc1.pdf]

Supplementary File

Title: Mortality and Infectious Adverse Events in Neutropenic Patients undergoing Gastrointestinal Endoscopic Procedures: A Systematic Review and Meta-Analysis

Authors: Janak Bahirwani, MD[1,8] Rishika Chugh MD[2], Ashley N. Tran MD[3], Amy Ogurick MD[4], Alyssa A. Grimshaw MSLIS, MPH[5], Maria Ciarleglio PhD[4], Yanhong Deng MPH[4], Badr Al-Bawardy MD4,6, Kenneth W. Hung MD, MS[4], Loren Laine MD[4,7]

1. Kadlec Regional Medical Center, Richland, Washington, USA
2. University of California, San Francisco, San Francisco, California, USA
3. St Luke's University Health Network, Bethlehem, Pennsylvania, USA
4. Yale School of Medicine, New Haven, Connecticut, USA
5. Harvey Cushing/John Hay Whitney Medical Library, Yale University, New Haven, Connecticut, USA
6. King Faisal Specialist Hospital, Riyadh, Saudi Arabia
7. VA Connecticut Health Care System, West Haven, Connecticut, USA
8. Elson S. Floyd College of Medicine at Washington State University Tri-cities campus

Appendix A: Reporting Guideline Checklists

PRISMA 2020 Main Checklist

Topic	No.	Item	Location where item is reported
**TITLE**			
**Title**	1	Identify the report as a systematic review.	LN1-2
**ABSTRACT**			
**Abstract**	2	See the PRISMA 2020 for Abstracts checklist	Appendix 1
**INTRODUCTION**			
**Rationale**	3	Describe the rationale for the review in the context of existing knowledge.	LN140-169
**Objectives**	4	Provide an explicit statement of the objective(s) or question(s) the review addresses.	LN1171-173
**METHODS**			
**Eligibility criteria**	5	Specify the inclusion and exclusion criteria for the review and how studies were grouped for the syntheses.	LN198-203
**Information sources**	6	Specify all databases, registers, websites, organisations, reference lists and other sources searched or consulted to identify studies. Specify the date when each source was last searched or consulted.	LN182-187
**Search strategy**	7	Present the full search strategies for all databases, registers and websites, including any filters and limits used.	Appendix B
**Selection process**	8	Specify the methods used to decide whether a study met the inclusion criteria of the review, including how many reviewers screened each record and each report retrieved, whether they worked independently, and if applicable, details of automation tools used in the process.	LN189-192
**Data collection process**	9	Specify the methods used to collect data from reports, including how many reviewers collected data from each report, whether they worked independently, any processes for obtaining or confirming data from study investigators, and if applicable, details of automation tools used in the process.	LN206-208
**Data items**	10a	List and define all outcomes for which data were sought. Specify whether all results that were compatible with each outcome domain in each study were sought (e.g. for all measures, time points, analyses), and if not, the methods used to decide which results to collect.	LN218-240
	10b	List and define all other variables for which data were sought (e.g. participant and intervention characteristics, funding sources). Describe any assumptions made about any missing or unclear information.	LN234-240
**Study risk of bias assessment**	11	Specify the methods used to assess risk of bias in the included studies, including details of the tool(s) used, how many reviewers assessed each study and whether they worked independently, and if applicable, details of automation tools used in the process.	LN209-215

Topic	No.	Item	Location where item is reported
**Effect measures**	12	Specify for each outcome the effect measure(s) (e.g. risk ratio, mean difference) used in the synthesis or presentation of results.	LN243-250
**Synthesis methods**	13a	Describe the processes used to decide which studies were eligible for each synthesis (e.g. tabulating the study intervention characteristics and comparing against the planned groups for each synthesis (item 5)).	LN243-250
	13b	Describe any methods required to prepare the data for presentation or synthesis, such as handling of missing summary statistics, or data conversions.	LN218-250
	13c	Describe any methods used to tabulate or visually display results of individual studies and syntheses.	LN218-250
	13d	Describe any methods used to synthesize results and provide a rationale for the choice(s). If meta-analysis was performed, describe the model(s), method(s) to identify the presence and extent of statistical heterogeneity, and software package(s) used.	LN218-250
	13e	Describe any methods used to explore possible causes of heterogeneity among study results (e.g. subgroup analysis, meta-regression).	LN252-256
	13f	Describe any sensitivity analyses conducted to assess robustness of the synthesized results.	LN252-256
**Reporting bias assessment**	14	Describe any methods used to assess risk of bias due to missing results in a synthesis (arising from reporting biases).	LN209-215
**Certainty assessment**	15	Describe any methods used to assess certainty (or confidence) in the body of evidence for an outcome.	LN252-256
**RESULTS**			
**Study selection**	16a	Describe the results of the search and selection process, from the number of records identified in the search to the number of studies included in the review, ideally using a flow diagram.	LN259-263
	16b	Cite studies that might appear to meet the inclusion criteria, but which were excluded, and explain why they were excluded.	Appendix C
**Study characteristics**	17	Cite each included study and present its characteristics.	LN263-286
**Risk of bias in studies**	18	Present assessments of risk of bias for each included study.	LN329-335
**Results of individual studies**	19	For all outcomes, present, for each study: (a) summary statistics for each group (where appropriate) and (b) an effect estimate and its precision (e.g. confidence/credible interval), ideally using structured tables or plots.	LN263-335
**Results of syntheses**	20a	For each synthesis, briefly summarise the characteristics and risk of bias among contributing studies.	LN263-335

Topic	No.	Item	Location where item is reported
	20b	Present results of all statistical syntheses conducted. If meta-analysis was done, present for each the summary estimate and its precision (e.g. confidence/credible interval) and measures of statistical heterogeneity. If comparing groups, describe the direction of the effect.	LN307-335
	20c	Present results of all investigations of possible causes of heterogeneity among study results.	LN307-335
	20d	Present results of all sensitivity analyses conducted to assess the robustness of the synthesized results.	LN307-335
**Reporting biases**	21	Present assessments of risk of bias due to missing results (arising from reporting biases) for each synthesis assessed.	LN338-344
**Certainty of evidence**	22	Present assessments of certainty (or confidence) in the body of evidence for each outcome assessed.	LN307-344
**DISCUSSION**			
**Discussion**	23a	Provide a general interpretation of the results in the context of other evidence.	LN347-404
	23b	Discuss any limitations of the evidence included in the review.	LN407-417
	23c	Discuss any limitations of the review processes used.	LN419-426
	23d	Discuss implications of the results for practice, policy, and future research.	LN428-432
**OTHER INFORMATION**			
**Registration and protocol**	24a	Provide registration information for the review, including register name and registration number, or state that the review was not registered.	LN176-179
	24b	Indicate where the review protocol can be accessed, or state that a protocol was not prepared.	LN176-179
	24c	Describe and explain any amendments to information provided at registration or in the protocol.	Available on PROSPERO
**Support**	25	Describe sources of financial or non-financial support for the review, and the role of the funders or sponsors in the review.	LN30
**Competing interests**	26	Declare any competing interests of review authors.	LN32-44
**Availability of data, code and other materials**	27	Report which of the following are publicly available and where they can be found: template data collection forms; data extracted from included studies; data used for all analyses; analytic code; any other materials used in the review.	Upon Request

From: Page MJ, McKenzie JE, Bossuyt PM, Boutron I, Hoffmann TC, Mulrow CD, et al. The PRISMA 2020 statement: an updated guideline for reporting systematic reviews. MetaArXiv. 2020, September 14. DOI: 10.31222/osf.io/v7gm2. For more information, visit: www.prisma-statement.org

PRISMA Abstract Checklist

Topic	No.	Item	Reported?
**TITLE**			
**Title**	1	Identify the report as a systematic review.	Yes
**BACKGROUND**			
**Objectives**	2	Provide an explicit statement of the main objective(s) or question(s) the review addresses.	Yes
**METHODS**			
**Eligibility criteria**	3	Specify the inclusion and exclusion criteria for the review.	Yes
**Information sources**	4	Specify the information sources (e.g. databases, registers) used to identify studies and the date when each was last searched.	Yes
**Risk of bias**	5	Specify the methods used to assess risk of bias in the included studies.	Yes
**Synthesis of results**	6	Specify the methods used to present and synthesize results.	Yes
**RESULTS**			
**Included studies**	7	Give the total number of included studies and participants and summarise relevant characteristics of studies.	Yes
**Synthesis of results**	8	Present results for main outcomes, preferably indicating the number of included studies and participants for each. If meta-analysis was done, report the summary estimate and confidence/credible interval. If comparing groups, indicate the direction of the effect (i.e. which group is favoured).	Yes
**DISCUSSION**			
**Limitations of evidence**	9	Provide a brief summary of the limitations of the evidence included in the review (e.g. study risk of bias, inconsistency and imprecision).	Yes
**Interpretation**	10	Provide a general interpretation of the results and important implications.	Yes
**OTHER**			
**Funding**	11	Specify the primary source of funding for the review.	Yes
**Registration**	12	Provide the register name and registration number.	Yes

MOOSE Checklist for Meta-analyses of Observational Studies

Item No	Recommendation	Reported on Page No
Reporting of background should include		
1	Problem definition	6-7
2	Hypothesis statement	6-7
3	Description of study outcome(s)	6-7
4	Type of exposure or intervention used	6-7
5	Type of study designs used	6-7
6	Study population	6-7
Reporting of search strategy should include		
7	Qualifications of searchers (eg, librarians and investigators)	7, Title page
8	Search strategy, including time period included in the synthesis and key words	7, Appendix B
9	Effort to include all available studies, including contact with authors	7-8
10	Databases and registries searched	7
11	Search software used, name and version, including special features used (eg, explosion)	7
12	Use of hand searching (eg, reference lists of obtained articles)	8
13	List of citations located and those excluded, including justification	Figure 1
14	Method of addressing articles published in languages other than English	7-8
15	Method of handling abstracts and unpublished studies	7-8
16	Description of any contact with authors	7-8
Reporting of methods should include		
17	Description of relevance or appropriateness of studies assembled for assessing the hypothesis to be tested	8-10
18	Rationale for the selection and coding of data (eg, sound clinical principles or convenience)	8-10
19	Documentation of how data were classified and coded (eg, multiple raters, blinding and interrater reliability)	8-10
20	Assessment of confounding (eg, comparability of cases and controls in studies where appropriate)	8-10
21	Assessment of study quality, including blinding of quality assessors, stratification or regression on possible predictors of study results	8-10
22	Assessment of heterogeneity	8-10
23	Description of statistical methods (eg, complete description of fixed or random effects models, justification of whether the chosen models account for predictors of study results, dose-response models, or cumulative meta-analysis) in sufficient detail to be replicated	8-10

24	Provision of appropriate tables and graphics	Table 1<br>Figure 2-5
Reporting of results should include		
25	Graphic summarizing individual study estimates and overall estimate	Table 1<br>Figure 2-5
26	Table giving descriptive information for each study included	Table 1
27	Results of sensitivity testing (eg, subgroup analysis)	Figure 2-5
28	Indication of statistical uncertainty of findings	10-16

**Item No**	**Recommendation**	**Reported on Page No**
Reporting of discussion should include		
29	Quantitative assessment of bias (eg, publication bias)	13
30	Justification for exclusion (eg, exclusion of non-English language citations)	16-17
31	Assessment of quality of included studies	13-15
Reporting of conclusions should include		
32	Consideration of alternative explanations for observed results	17
33	Generalization of the conclusions (ie, appropriate for the data presented and within the domain of the literature review)	17
34	Guidelines for future research	17
35	Disclosure of funding source	1

From: Stroup DF, Berlin JA, Morton SC, et al, for the Meta-analysis Of Observational Studies in Epidemiology (MOOSE) Group. Meta-analysis of Observational Studies in Epidemiology. A Proposal for Reporting. *JAMA*. 2000;283(15):2008-2012. doi: 10.1001/jama.283.15.2008.

Appendix B: Search Strategies

Ovid Embase

1 exp digestive tract endoscopy/
2 (endoscop* adj3 (digestive or gastrointestinal)).tw,kw.
3 (balloon enteroscop* or (push and pull endoscop*) or double balloon endoscop* or single balloon endoscop* or Colonoscop* or Sigmoidoscop* or Proctosigmoidoscop* or Duodenoscop*).tw,kw.
4 (Esophagoscop* or Gastroscop* or Proctoscop* or strip biosp* or esophagogastroduodenoscop* or endoscopic ultrasound* or percutaneous endoscopic gastrostom*).tw,kw.
5 (Endoscopic adj (Mucosal or submucosal) adj3 (Resection* or Dissection*)).tw,kw.
6 (endoscop* adj3 retrograd* adj3 cholangio*).tw,kw.
7 (EGD or ERCP or EUS or PEG).ti,ab.
8 1 or 2 or 3 or 4 or 5 or 6 or 7
9 exp Leukopenia/
10 (neutropaeni* or neutropeni*).tw,kw.
11 (neutrophil* adj3 (dysfunction* or disease* or disorder*)).tw,kw.
12 (leukopeni* or leukocytopeni* or agranulocytoses or agranulocytosis or granulocytopeni* or lymphopeni* or lymphocytopeni*).tw,kw.
13 (pancytopeni* or cytopeni* or bicytopeni*).tw,kw.
14 9 or 10 or 11 or 12 or 13
15 8 and 14
16 exp animal/
17 exp animal/ and exp human/
18 16 not 17
19 15 not 18
20 exp embryo/ or exp infant/ or exp juvenile/
21 (exp embryo/ or exp infant/ or exp juvenile/) and exp adult/
22 20 not 21
23 19 not 22
24 limit 23 to conference abstracts
25 23 not 24
26 limit 25 to english language

Ovid MEDLINE(R) ALL

1 exp Endoscopy, Gastrointestinal/
2 (endoscop* adj3 (digestive or gastrointestinal)).tw,kf.

3 (balloon enteroscop* or (push and pull endoscop*) or double balloon endoscop* or single balloon endoscop* or Colonoscop* or Sigmoidoscop* or Proctosigmoidoscop* or Duodenoscop*).tw,kf.
4 (Esophagoscop* or Gastroscop* or Proctoscop* or strip biosp* or esophagogastroduodenoscop* or endoscopic ultrasound* or percutaneous endoscopic gastrostom*).tw,kf.
5 (Endoscopic adj (Mucosal or submucosal) adj3 (Resection* or Dissection*)).tw,kf.
6 (endoscop* adj3 retrograd* adj3 cholangio*).tw,kf.
7 (EGD or ERCP or EUS or PEG).ti,ab.
8 or/1-7
9 exp Leukopenia/
10 (neutropaeni* or neutropeni*).tw,kf.
11 (neutrophil* adj3 (dysfunction* or disease* or disorder*)).tw,kf.
12 (leukopeni* or leukocytopeni* or agranulocytoses or agranulocytosis or granulocytopeni* or lymphopeni* or lymphocytopeni*).tw,kf.
13 (pancytopeni* or cytopeni* or bicytopeni*).tw,kf.
14 or/9-13
15 8 and 14
16 exp animal/
17 exp animal/ and exp human/
18 16 not 17
19 15 not 18
20 exp child/ or exp infant/
21 (exp child/ or exp infant/) and exp adults/
22 20 not 21
23 19 not 22
24 limit 23 to english language

Web of Science

#1 TS=(endoscop* near/3 (digestive or gastrointestinal)) or TS=("balloon enteroscop*" or "push and pull endoscop*" or "double balloon endoscop*" or "single balloon endoscop*" or Colonoscop* or Sigmoidoscop* or Proctosigmoidoscop* or Duodenoscop*) or TS=(Esophagoscop* or Gastroscop* or Proctoscop* or "strip biosp*" or sophagogastroduodenoscop* or "endoscopic ultrasound*" or "percutaneous endoscopic gastrostom*") or TS=(Endoscopic near/1 (Mucosal or submucosal) near/3 (Resection* or Dissection*)) or TS=(endoscop* near/3 retrograd* near/3 cholangio*) or TS=(EGD or ERCP or EUS or PEG)
#2 TS=(neutropaeni* or neutropeni*) or TS=(neutrophil* near/3 (dysfunction* or disease* or disorder*)) or TS=(leukopeni* or leukocytopeni* or agranulocytoses or agranulocytosis or granulocytopeni* or lymphopeni* or lymphocytopeni*) or TS=(pancytopeni* or cytopeni* or bicytopeni*)
#3 #1 and #2

Scopus

(TITLE-ABS-KEY (neutropaeni* OR neutropeni*) OR TITLE-ABS-KEY (neutrophil* W/3 (dysfunction* OR disease* OR disorder*)) OR TITLE-ABS-KEY (leukopeni* OR leukocytopeni* OR agranulocytoses OR agranulocytosis OR granulocytopeni* OR lymphopeni* OR lymphocytopeni*) OR TITLE-ABS-KEY (pancytopeni* OR cytopeni* OR bicytopeni*)) AND (TITLE-ABS-KEY (endoscop* W/3 (digestive OR gastrointestinal)) OR TITLE-ABS-KEY ("balloon enteroscop*" OR "push and pull endoscop*" OR "double balloon endoscop*" OR "single balloon endoscop*" OR colonoscop* OR sigmoidoscop* OR proctosigmoidoscop* OR duodenoscop*) OR TITLE-ABS-KEY (esophagoscop* OR gastroscop* OR proctoscop* OR "strip biosp*" OR sophagogastroduodenoscop* OR "endoscopic ultrasound*" OR "percutaneous endoscopic gastrostom*") OR TITLE-ABS-KEY (endoscopic W/1 (mucosal OR submucosal) W/3 (resection* OR dissection*)) OR TITLE-ABS-KEY (endoscop* W/3 retrograd* W/3 cholangio*) OR TITLE-ABS-KEY (egd OR ercp OR eus OR peg)) AND (LIMIT-TO (LANGUAGE , "English"))

Cochrane Library

#1 (endoscop* near/3 (digestive or gastrointestinal)):ti,ab or ("balloon enteroscop*" or "push and pull endoscop*" or "double balloon endoscop*" or "single balloon endoscop*" or Colonoscop* or Sigmoidoscop* or Proctosigmoidoscop* or Duodenoscop*):ti,ab or (Esophagoscop* or Gastroscop* or Proctoscop* or "strip biosp*" or sophagogastroduodenoscop* or "endoscopic ultrasound*" or "percutaneous endoscopic gastrostom*"):ti,ab or (Endoscopic near/1 (Mucosal or submucosal) near/3 (Resection* or Dissection*)):ti,ab or (endoscop* near/3 retrograd* near/3 cholangio*):ti,ab or (EGD or ERCP or EUS or PEG):ti,ab

#2 (neutropaeni* or neutropeni*):ti,ab or (neutrophil* near/3 (dysfunction* or disease* or disorder*)):ti,ab or (leukopeni* or leukocytopeni* or agranulocytoses or agranulocytosis or granulocytopeni* or lymphopeni* or lymphocytopeni*):ti,ab or (pancytopeni* or cytopeni* or bicytopeni*):ti,ab

#3 #1 and #2

PubMed

(neutropaeni* or neutropeni* or neutrophil* dysfunction* or neutrophil* disease* or neutrophil* disorder* or leukopeni* or leukocytopeni* or agranulocytoses or agranulocytosis or granulocytopeni* or lymphopeni* or lymphocytopeni* or pancytopeni* or cytopeni* or bicytopeni*) AND ((digestive endoscop*[Title/Abstract] OR gastrointestinal endoscop*[Title/Abstract] OR "balloon enteroscop*"[Title/Abstract] OR "push and pull endoscop*"[Title/Abstract] OR "double balloon endoscop*"[Title/Abstract] OR "single balloon endoscop*"[Title/Abstract] OR Colonoscop*[Title/Abstract] OR Sigmoidoscop*[Title/Abstract] OR Proctosigmoidoscop*[Title/Abstract] OR Duodenoscop*[Title/Abstract] OR Esophagoscop*[Title/Abstract] OR Gastroscop*[Title/Abstract] OR Proctoscop*[Title/Abstract] OR "strip biosp*"[Title/Abstract] OR

sophagogastroduodenoscop*[Title/Abstract] OR "endoscopic ultrasound*"[Title/Abstract] OR "percutaneous endoscopic gastrostom*"[Title/Abstract] OR endoscopic mucosal resection*[Title/Abstract] OR endoscopic submucosal resection*[Title/Abstract] OR endoscopic submucosal dissection*[Title/Abstract] OR endoscopic mucosal dissection*[Title/Abstract] OR endoscop* retrograd* cholangio*[Title/Abstract] OR EGD[Title/Abstract] OR ERCP[Title/Abstract] OR EUS[Title/Abstract] OR PEG[Title/Abstract]))

Google Scholar

neutropeni* gastrointestinal endoscopy

Appendix C: Excluded Studies Table

First Author Last Name	Year	Title	Journal	Exclusion Reason
Abu-Sbeih	2018	Mo1107 PREDICTORS OF OVERALL SURVIVAL IN CANCER PATIENTS WHO HAD ENDOSCOPIC EVALUATION IN THE SETTING OF NEUTROPENIA AND THROMBOCYTOPENIA	Gastrointestinal Endoscopy	Not enough details
Abu-Sbeih	2019	Neutropenic Colitis - Clinical Features, Treatment and Outcomes	Gastroenterology	Wrong outcomes
Agarwal	2016	Sepsis in a case of severe neutropenia unresponsive to G-CSF; a rare sequelae of chronic hepatitis C	American Journal of Gastroenterology	Wrong study design
AlAshgar	2009	Defecation of a "colon cast" as a rare presentation of acute graft-versus-host disease	Annals of Saudi Medicine	Wrong study design
Al-Azzawi	2016	ERCP for septic cholangitis through a peg tube, a life-saving procedure using an alternative access route to approach the biliary tree when the regular ERCP fails	American Journal of Gastroenterology	Wrong study design
Allen	2013	Efficacy and safety of treatment of hepatitis c in patients with inflammatory bowel disease	Clinical Gastroenterology and Hepatology	Wrong outcomes
Apanasenko	2018	A case of cytomegalovirus-induced pancytopenia with CMV colitis	HemaSphere	Wrong study design
Arai	1990	[Cyclic neutropenia complicated of non-Hodgkin lymphoma]	Rinsho Ketsueki - Japanese Journal of Clinical Hematology	Wrong study design
Armstrong	2018	Protracted severe systemic cytomegalovirus disease in an immunosuppressed patient with ulcerative colitis	Frontline Gastroenterology	Wrong study design
Auguste	1986	Postchemotherapy esophagitis: The endoscopic diagnosis and its impact on survival	Journal of Surgical Oncology	Wrong outcomes
Banerjee	2017	Role of emergency endoscopic decompression of colon in a case of compartment like syndrome of left lower limb secondary to severe acute pancreatitis	Surgical Endoscopy and Other Interventional Techniques	Wrong study design

Bernal	2016	Recurrent right-sided pleural effusion secondary to a pancreatic-pleural fistula: An endoscopic solution to a rare complication	American Journal of Gastroenterology	Wrong study design
Bernal	2017	Recurrent pleural effusion secondary to a pancreatic-pleural fistula treated endoscopically	American Journal of Case Reports	Wrong study design
Bitton	2016	Cytomegalovirus (CMV) colitis trigerring inflammatory bowel disease (IBD) in an immunocompetent adult: A case report and review of the literature	Canadian Journal of Gastroenterology and Hepatology. Conference	Wrong study design
Bolon	2017	Hemophagocytic lymphohistiocytosis (HLH), a rare and catastrophic cause of GI Bleeding	South African Gastroenterology Review	Wrong study design
Candiani	2019	Black esophagus: Acute esophageal necrosis syndrome: Case report	Italian Journal of Medicine	Wrong study design
Carroccio	2003	Autoimmune enteropathy and colitis in an adult patient	Digestive Diseases and Sciences	Wrong study design
Chen	2019	Additional Radiotherapy for Superficial Esophageal Cancer Following Endoscopic Submucosal Dissection (ESD): A Single Center Retrospective Study	Nanoscience and Nanotechnology Letters	Wrong intervention
Chisti	2013	Dasatinib-induced haemorrhagic colitis in chronic myeloid leukaemia (CML) in blast crisis	BMJ Case Reports.	Wrong study design
Choi	2011	A case of Crohn's disease with improvement after azathioprine-induced pancytopenia	Case Reports in Gastroenterology	Wrong study design
Chong	2005	Human immunodeficiency virus and endoscopy: Experience of a general hospital in Singapore	Journal of Gastroenterology and Hepatology (Australia)	Wrong patient population
Cortes-Flores	2015	Long-term outcome after percutaneous endoscopic gastrostomy in geriatric Mexican patients	Geriatrics and Gerontology International	Wrong patient population
Crudeli	2017	Infection following lower gastrointestinal endoscopy: silent risk or non-event?	Endoscopy	Wrong study design
Docker	2010	[Gastric neuroendocrine tumors in a woman with systemic lupus erythematosus]	Deutsche Medizinische Wochenschrift	Wrong study design
Dworkin	1992	The safety and efficacy of gi endoscopy in patients with acute-leukemia - a review of 27 cases	International Journal of Oncology	Wrong outcomes

Elden	2016	A case of inflammatory bowel disease and a rare lymphoma on azathioprine therapy	American Journal of Gastroenterology	Wrong study design
Elfeki	2014	Abatacept use in graft-versus-host disease after orthotopic liver transplantation: A case report	Transplantation Proceedings	Wrong study design
ElRafei	2018	Splenomegaly with a side of sinister portal hypertension	American Journal of Tropical Medicine and Hygiene	Wrong study design
Endoscopy	1999	Infection Control During Gastrointestinal Endoscopy	Gastrointestinal Endoscopy	Wrong study design
Forbes	1995	A prospective study of screening upper gastrointestinal (GI) endoscopy prior to and after bone marrow transplantation (BMT)	Aust N Z J Med	Not enough details
Gorschluter	2008	Endoscopy in patients with acute leukaemia after intensive chemotherapy	Leukemia Research	Wrong patient population
Greene	1974	Esophagoscopy as a source of Pseudomonas aeruginosa sepsis in patients with acute leukemia: the need for sterilization of endoscopes	Gastroenterology	Wrong study design
Han	2019	The Risk of Infectious Adverse Events after Eus-Guided Fine Needle Biopsy	Gastrointestinal Endoscopy	Wrong patient population
Han	2019	Mo1060 THE RISK OF INFECTIOUS ADVERSE EVENTS AFTER EUS-GUIDED FINE NEEDLE BIOPSY	Gastrointestinal	Not enough details
Hefazi	2016	Safety and efficacy of fecal microbiota transplantation for recurrent clostridium infection in patients with hematologic malignancies	Blood	Wrong patient population
Hermes	2011	[Non-small-cell carcinoma of the lung with invasive Aspergillus infection after chemotherapy]	Deutsche Medizinische Wochenschrift	Non-English language
Hess	1997	Botryomycosis causing giant ulcers of the esophagus: A case report	Gastrointestinal Endoscopy	Wrong study design
Jafri	2009	Utility and safety of endoscopic procedures in neutropenic patients with gastrointestinal bleeding	Gastrointestinal Endoscopy	Not enough details
Janssen	2004	Frequency of bacteremia after linear EUS of the upper GI tract with and without FNA	Gastrointestinal Endoscopy	Wrong patient population

Jiang	2012	Endoscopic stenting and concurrent chemoradiotherapy for advanced esophageal cancer: a case-control study	World Journal of Gastroenterology	Wrong patient population
Kara	2016	Survival After Percutaneous Endoscopic Gastrostomy in Older Adults With Neurologic Disorders	Nutrition in Clinical Practice	Wrong patient population
Khan	2004	Emerging bacterial resistance patterns in febrile neutropenic patients: experience at a tertiary care hospital in Pakistan	Journal of Pakistan	Wrong patient population
Leharova	2019	Incidence of blood stream infection (BSI) associated with endoscopic retrograde cholangiopancreatography in a tertiary hospital; 3 years prospective surveillance study	Antimicrobial Resistance and Infection Control.	Wrong patient population
Lorenz	1996	[Antibiotic prophylaxis using cefuroxime in bile duct endoscopy]	Deutsche Medizinische Wochenschrift	Non-English language
Nagata	2011	Diagnostic value of antigenemia assay for cytomegalovirus gastrointestinal disease in immunocompromised patients	World Journal of Gastroenterology	Wrong outcomes
Nevah	2014	Transnasal PEG tube placement in patients with head and neck cancer	Gastrointestinal Endoscopy	Not enough details
Otaki	2014	Sa1488 Retrospective Review of 100 Endoscopic Evaluations of Patients on Bone Marrow Transplant Service for GVHD	Gastrointestinal Endoscopy	Not enough details
Perez	2002	Endoscopy in patients receiving radiation therapy to the thorax	Digestive Diseases and Sciences	Wrong patient population
Rajan	2020	Tu1078 DOES THROMBOCYTOPENIA PREDICT POOR OUTCOME AFTER ENDOSCOPY FOR EVALUATION OF GRAFT-VERSUS-HOST DISEASE IN â€¦	Gastrointestinal Endoscopy	Not enough details
Ross	2013	Endoscopy in Hematologic Malignancies	Gastrointestinal Endoscopy in the Cancer Patient	Wrong study design
Soylu	2005	Overt gastrointestinal bleeding in haematologic neoplasms	Digestive and Liver Disease	Wrong outcomes

Tong	2014	Endoscopy in Neutropenic and/or Thrombocytopenic Patients: Review of Current Evidence and Development of Clinical Recommendations: 1842	Official journal of the American College of Gastroenterology| ACG	Wrong study design
Tong	2015	Endoscopy in neutropenic and/or thrombocytopenic patients	World Journal of Gastroenterology	Wrong study design
Tsirigotis	2008	Keratinocyte growth factor is effective in the prevention of intestinal mucositis in patients with hematological malignancies treated with high-dose chemotherapy and autologous hematopoietic SCT: A video-capsule endoscopy study	Bone Marrow Transplantation	Wrong patient population
Wheeler	1987	Esophagitis in the immunocompromised host: role of esophagoscopy in diagnosis	Reviews of Infectious Diseases	Not enough details
Yamazaki	2016	Impact of prophylactic percutaneous endoscopic gastrostomy tube placement on treatment tolerance in head and neck cancer patients treated with cetuximab plus radiation	Japanese Journal of Clinical Oncology	Wrong outcomes
Ye	2017	Therapeutic ERCP safely performed in patients with leukemia: A single center experience	Journal of Digestive Diseases	Not enough details

Appendix D: Risk of bias

Study	Selection (0-3)	Comparability (0-2)	Outcomes (0-3)	Total score	Overall risk of bias
Abu-Sbeih et al.	4	2	3	9	Low
Isenberg et al.	4	2	3	9	Low
Kaw et al.	3	1	2	6	High
Liu et al.	3	1	3	7	Low
Shin et al.	4	2	3	9	Low
Vishny et al.	4	2	2	8	Low
