# Supplementary material for: Mortality and Infectious Adverse Events in Neutropenic Patients Undergoing Gastrointestinal Endoscopic Procedures: A Systematic Review and Meta-Analysis
Source: Gastro Hep Adv. 2026 May 8;5(8):100994. doi: 10.1016/j.gastha.2026.100994 (PMC13264052; doi:10.1016/j.gastha.2026.100994)
Supplement: Extended PDF [file mmc2.pdf]

Gastro Hep Advances 2026;5:100994

SYSTEMATIC REVIEWS AND META-ANALYSIS

Mortality and Infectious Adverse Events in Neutropenic Patients Undergoing Gastrointestinal Endoscopic Procedures: A Systematic Review and Meta-Analysis

Janak Bahirwani,[1,2] Rishika Chugh,[3] Ashley N. Tran,[4] Amy Ogurick,[5] Alyssa A. Grimshaw,[6] Maria Ciarleglio,[7] Yanhong Deng,[7] Badr Al-Bawardy,[8,9] Kenneth W. Hung,[5] and Loren Laine[8,10]

[1]Department of Gastroenterology, Kadlec Regional Medical Center, Richland, Washington; [2]Department of Gastroenterology, Elson S. Floyd College of Medicine, Washington State University Tri-Cities Campus, Richland, Washington; [3]Department of Gastroenterology, University of California San Francisco, San Francisco, California; [4]Department of Gastroenterology, St Luke's University Health Network, Bethlehem, Pennsylvania; [5]Division of Digestive Health, Yale School of Medicine, New Haven, Connecticut; [6]Yale Cushing/Whitney Medical Library Division, Harvey Cushing/John Hay Whitney Medical Library, Yale University, New Haven, Connecticut; [7]Division of Analytical Sciences, Yale School of Medicine, New Haven, Connecticut; [8]Division of Digestive Health, Yale School of Medicine, New Haven, Connecticut; [9]Division of Digestive Health, King Faisal Specialist Hospital, Riyadh, Saudi Arabia; and [10]VA Connecticut Health Care System, West Haven, Connecticut

BACKGROUND AND AIMS: The safety of gastrointestinal endoscopy in neutropenic patients remains unestablished. Gastrointestinal and infectious disease society guidelines indicate that infectious adverse events are increased after endoscopy in neutropenic patients. We performed a systematic review and meta-analysis to assess the safety of endoscopy in neutropenic patients. **METHODS:** Cochrane Library, Embase, Google Scholar, MEDLINE, PubMed, Scopus, and Web of Science were searched through September 2025 for studies in neutropenic patients (absolute neutrophil count <1000 cells/μL) undergoing endoscopy with outcome data on infections, mortality, or fever. Conference abstracts from January 2017 to September 2025 were also searched. Two reviewers independently identified studies meeting inclusion criteria, performed data extraction, and assessed risk of bias. Coprimary outcomes were 30-day infection-related mortality and infectious adverse events within 7 days of endoscopy. Secondary outcomes were new bacteremia and new fever within 7 days of endoscopy. Random-effects meta-analyses were performed. **RESULTS:** Six cohort studies met eligibility criteria (N = 1241 patients). Pooled incidence was 0.0% (95% confidence interval [CI] 0.00%–0.03%; $I^2 = 0.0\%$) for infection-related mortality and 7.3% (95% CI 0.00%–25.57%; $I^2 = 98.6\%$) for infectious adverse events, which consisted of bacteremia (2.4% [95% CI 0.03%–6.99%; $I^2 = 87.3\%$]) and new fever (4.8% [95% CI 0.00%–9.74%; $I^2 = 99\%$]). Preprocedural antibiotic use did not reduce infectious adverse events or bacteremia as compared to no antibiotic use: odds ratio = 2.97, 95% CI 0.47–18.67. **CONCLUSION:** Infection-related mortality within 30 days was 0% and infectious adverse events within 7 days occurred in 7.3% of neutropenic patients undergoing endoscopy. These findings suggest endoscopy maybe safely performed in appropriately selected neutropenic patients.

Keywords: Neutropenia; Endoscopy; Immunosuppression; Infection

Introduction

The safety of endoscopy in patients with neutropenia is unclear. The American Society for Gastrointestinal Endoscopy guidelines from 2015 state "Patients with severe neutropenia (absolute neutrophil count [ANC] <500 cells/μL) and advanced hematologic malignancies are at increased risk for bacteremia and sepsis after gastrointestinal (GI) endoscopy. The protective effect of prophylactic antibiotics in this patient population has not been well studied. However, this practice seems logical, especially in patients undergoing endoscopic procedures that are associated with a high risk of bacteremia."[1] The British Society of Gastroenterology guidelines indicate that "neutropenia predisposes to sepsis after endoscopy" and that afebrile patients with ANC $<0.5 \times 10^9$/L should be offered antibiotic prophylaxis for procedures known to be associated with a high risk of bacteremia.[2] In contrast, the Infectious Diseases Society of America guidelines regarding neutropenic patients with cancer state that "diagnostic endoscopy rarely causes bacteremia."[3] Other studies state it is likely safe to proceed with endoscopy at varying levels of neutropenia.[3,4]

A systematic review from 2015 explored the safety of endoscopy in neutropenic cancer patients.[5] They identified only 3 studies providing data on infectious adverse events; 2 examined only pediatric populations,[6,7] while the third study examined only patients with leukemia and did not define the degree of neutropenia.[8] Based on these very limited data, the authors suggested it is safe to perform

***Abbreviations used in this paper:* ANC, absolute neutrophil count; EGD, esophagogastroduodenoscopy; GI, gastrointestinal; PEG, percutaneous endoscopic gastrostomy.**

Most current article

© 2026 The Authors. Published by Elsevier Inc. on behalf of American Gastroenterological Association Institute. This is an open access article under the CC BY license (http://creativecommons.org/licenses/by/4.0/).
2772-5723
https://doi.org/10.1016/j.gastha.2026.100994

endoscopy in neutropenic patients, although prophylactic antibiotic administration should be considered in some cases. Since the 2015 systematic review and the aforementioned guidelines from 2009 to 2015, additional studies have become available that should allow us to improve the precision of outcome estimates and potentially alter conclusions.

A recent systematic review by Loganathan et al[9] identified 4 studies with neutropenic patients,[4,8,10,11] and reported a pooled infectious adverse event rate of 6.8%. However, this review searched a limited number of databased and the degree of neutropenia was not specified for one of these studies. Since that publication, we identified additional studies to allow for more precise outcome estimates.

We therefore performed an updated, comprehensive systematic review including searches of additional databases to assess the safety of endoscopy in adult patients with neutropenia and the utility of antibiotic prophylaxis.

Methods

This systematic review and meta-analysis was conducted according to the Preferred Reporting Items for Systematic Reviews and Meta-Analyses and Meta-analysis of Observational Studies in Epidemiology guidelines (Supplementary Appendix A).[12,13] The study protocol was registered a priori on the International Prospective Register of Systematic Reviews (CRD42020155664). Institutional review board approval was not required for this meta-analysis because it analyzed only publicly available data from previously published studies and did not directly involve human participants.

Data Sources and Search Strategy

Bibliographic databases (Cochrane Library, Google Scholar, Ovid Embase, Ovid MEDLINE, PubMed, Scopus, Web of Science Core Collection) were searched through September 9, 2025, for studies in neutropenic patients (ANC $<$1000 cells/μL) undergoing endoscopy. An extensive search strategy was developed by 2 authors, including a medical librarian and reviewed by a second librarian using Peer Review of Electronic Search Strategies.[14] The search was restricted to English language citations, human subjects, and adults (Supplementary Appendix B: Search Strategies).

Titles and abstracts were independently reviewed by 3 reviewers (J.B., A.T., R.C.). Any citation considered potentially relevant underwent dual independent full-text review. Disagreements regarding study selection were resolved by consensus. Two authors (B.A., K.H.) served as final arbiters in the event of unresolved disagreement. Conference abstracts from Digestive Disease Week, American College of Gastroenterology Annual Scientific Meeting, and United European Gastroenterology Week from January 2017 to September 2025 also were searched by 3 reviewers (J.B., R.C., A.O.).

Study Selection

Studies were limited to cohort studies (single arm or comparative) and randomized controlled trials. The population was limited to adult patients ($\geq$18 years) with ANC less than 1000 cells/μL undergoing endoscopy. Because our outcomes are incidences, case-control studies, case series and case reports were excluded as these do not allow determination of incidences. Unpublished data were excluded with the exception of conference abstracts between January 2017 and September 2025.

Data Extraction and Quality Assessment

Two reviewers independently (R.C./A.T., R.C./A.O., R.C./J.B., A.T./J.B.) extracted data and performed risk-of-bias assessment for each study using a standardized form. Disagreements were resolved by consensus. If consensus was not reached, a third independent reviewer (A.T., A.O., R.C.) served as final arbiter. Authors were not contacted for additional unpublished data. The Newcastle-Ottawa Scale for cohort studies was used to evaluate methodological quality of studies.[10] This scale assigns a maximum of 9 stars for lowest risk of bias in the following 3 domains: selection of study groups (4 stars); comparability of study groups (2 stars); and ascertainment of outcomes (3 stars). Risk of bias was determined by comparing the total Newcastle-Ottawa Scale scores categorized into the following 3 groups: very high risk-of-bias-0-3; high risk-of-bias-4-6; and low risk-of-bias-7-9.[15]

Study Outcomes

Primary outcomes included infection-related mortality within 30 days and infectious adverse events within 7 days following endoscopy. Secondary outcomes were new bacteremia and new-onset fever within 7 days of endoscopy. We also assessed the outcome of infectious adverse events in patients receiving and not receiving antibiotics prior to endoscopy. Characteristics of study populations included age, sex, race, history of malignancy (solid or hematologic), recent stem cell transplant, immunosuppressant use, and neutropenia. Immunosuppressants were defined as biologic therapies, chemotherapy, and steroids.

Procedural details including type of procedure (esophagogastroduodenoscopy [EGD], colonoscopy, sigmoidoscopy, enteroscopy, endoscopic retrograde cholangiopancreatography, endoscopic ultrasound, percutaneous endoscopic gastrostomy [PEG] tube placement) and procedural interventions (biopsy, hemostatic therapy, and sphincterotomy) were extracted. Data on use of antibiotics immediately prior to or periprocedure, when available, were recorded. Indications for procedures were separated into the following categories: anorexia/weight loss; nausea/vomiting; diarrhea; overt GI bleeding or anemia; dysphagia/odynophagia; abdominal pain; and other. Endoscopic and histologic findings were recorded as the following: ulcer; varices; other bleeding lesion; graft vs host disease; cytomegalovirus; other viral infection; candidiasis; other fungal infection; and *Clostridium difficile*.

Infection details were extracted, including the timing of infection in relation to the procedure and how each study defined an infection. We defined an infectious adverse event as new fever, new bacteremia, or PEG tube site infection following endoscopy with fever and bacteremia not present prior to endoscopy.

Data Synthesis and Statistical Analysis

Random-effects model meta-analysis was employed using the inverse variance method. A random-effects model was chosen because marked heterogeneity across studies was anticipated given the likely great variability in populations,

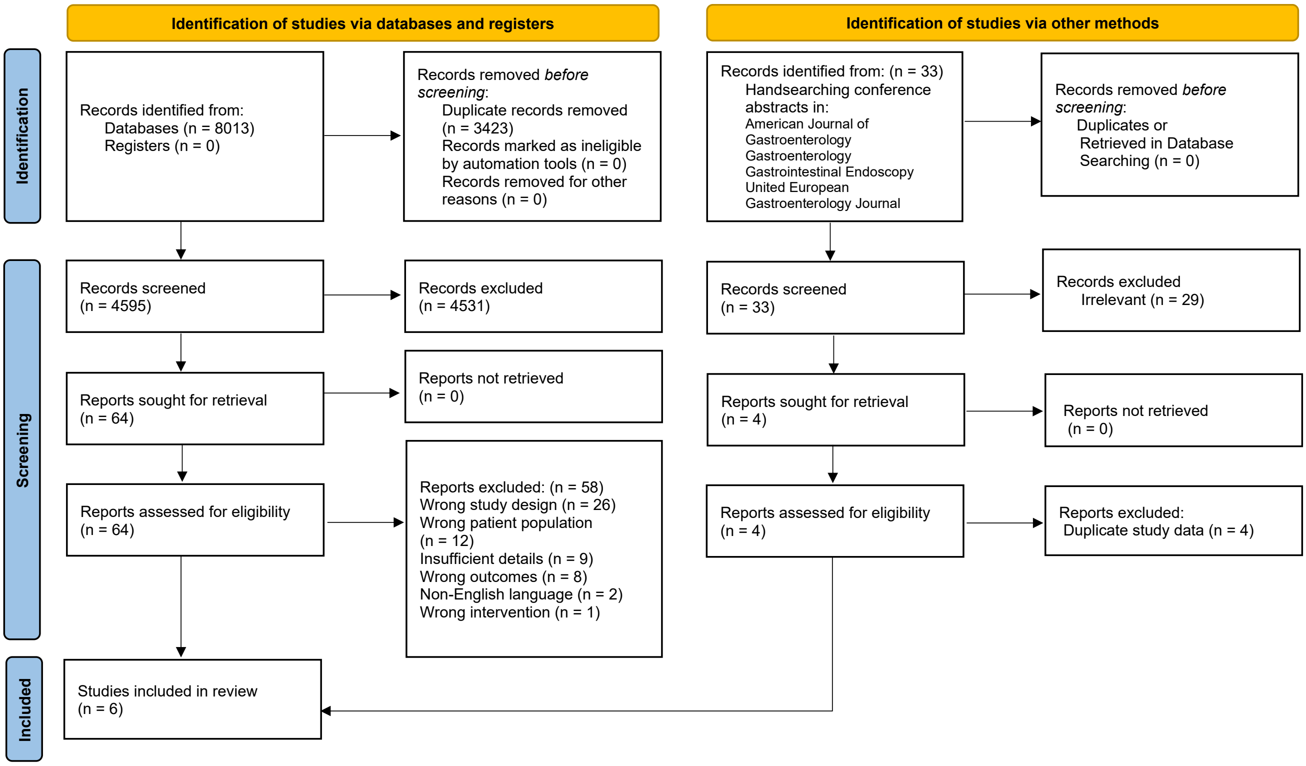

Adapted from: Page MJ, McKenzie JE, Bossuyt PM, Boutron I, Hoffmann TC, Mulrow CD, et al. The PRISMA 2020 statement: an updated guideline for reporting systematic reviews. BMJ 2021;372: n71. doi: 10.1136/bmj. n71. For more information, visit: http://www.prisma-statement.org/

Figure 1. PRISMA flow diagram summarizing study selection. PRISMA, Preferred Reporting Items for Systematic Reviews and Meta-Analyses.

endoscopic procedures, and assessment of infections; a prior meta-analysis showed marked statistical heterogeneity for our primary outcome.[9] Proportions were transformed using Freeman-Tukey double arcsine transformation for variance stabilization. Due to marked variation in sample size and several zero numerators in individual studies, a generalized linear mixed model analysis was also performed for sensitivity assessment; this yielded similar results to the Freeman-Tukey double arcsine transformation. However, particularly when evaluating new fever, the generalized linear mixed model analysis yielded an upper bound of zero for the confidence interval (CI). We therefore opted for the Freeman-Tukey double arcsine transformation.

The final pooled proportions and CIs were back-transformed for ease of interpretation. Heterogeneity between studies was assessed using the I^2 statistic and the *Q test* with substantial heterogeneity defined as an $I^2 > 50\%$ or $P < .10$, respectively. Publication bias was evaluated using visual inspection of a funnel plot for asymmetry. Forest plots were constructed to summarize pooled estimates and to graphically display results from individual studies.

Results

A total of 4595 unique citations were identified from the systematic search, and 4 citations were identified through hand search of conference abstracts. After title and abstract review, 64 studies were selected for full-text review. Studies that were reviewed in full text review but not included with specific reason for exclusion are provided in Supplementary Appendix C. A total of 6 studies met the inclusion criteria (Figure 1).[4,10,11,16–18] No additional relevant studies were identified in our review of conference abstracts. All the 6 studies were single-arm cohorts. While Liu et al[17] identified as a 2-armed cohort study (including patients with and without gastrointestinal graft vs host disease), for purposes of our study this was interpreted as a single-arm cohort as all patients included in our analysis had undergone hematopoietic stem cell transplant and were neutropenic. The 6 studies included 1241 procedures performed on neutropenic patients (range 10–675) (Table). The follow-up period for infectious adverse events related to endoscopy was 1–7 days with a median of 5 days. The follow-up period for mortality was 3–30 days with a median of 30 days.

Population Characteristics

Data on malignancy diagnosis were available in all the 6 studies; 88% (N = 1089) of procedures were performed in patients carrying a cancer diagnosis as follows: 812 with a hematologic malignancy and 246 with a solid malignancy.[4,10,11,16–18] For 31 procedures, the type of malignancy (hematologic vs solid) was unknown. Five of the 6 studies evaluated the impact of immunosuppressant use; 37.9% (N = 467) of procedures in these studies were performed on patients receiving immunosuppressants.[4,10,11,17,18]

Table. Study and Population Characteristics

Study	Abu-Sbeih, 2019	Isenberg, 2022	Kaw, 1993	Liu, 2013	Shin, 2022	Vishny, 1994
Location	United States	Israel	United States	United States	South Korea	United States
Study design	Cohort	Cohort	Cohort	Case-control	Cohort	Cohort
Total procedures performed on neutropenic patients	675	167	10	40	313	36
Mean age (y)	57.9	56.0	37.1	43.8	49.0	31.5
Male (%)	59	45.5	45	54	56	44
Duration follow-up (d)						
For infectious adverse events	7	2	–	7	7	3
For infection-related mortality	30	7	–	–	–	3
Malignancy diagnosis, n (%)	675 (100)	21 (12)	10 (100)	40 (100)	307 (98)	36 (100)
Solid	246 (36)	–	–	0 (0)	0 (0)	0 (0)
Hematologic	429 (64)	–	–	40 (100)	307 (100)	36 (100)
Stem cell transplant recipient, n (%)	147 (22)	34 (20)	10 (100)	40 (100)	74 (24)	36 (100)
Immunosuppressant recipient, n (%)	184 (27)	143 (73)	–	40 (100)	64 (20)	36 (100)
ANC at time of procedure, n (%)		<1000 but not subclassified	<1000 but not subclassified	<1000 but not subclassified		
500–1000 cells/μL	420 (62)	–	–	–	139 (44)	0 (0)
<500 cells/μL	255 (38)	–	–	–	174 (56)	36 (100)
Antibiotic use, n (%)	485 (72)	94 (56)	7 (70)	–	300 (96)	36 (100)

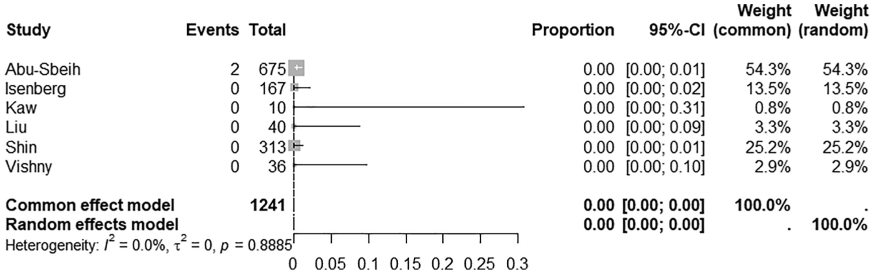

Figure 2. Forest plot of incidence of infection-related mortality.

Data on history of stem cell transplant were available in all the 6 studies; 27.4% (N = 341) of procedures were performed on patients who were stem cell transplant recipients. All studies had patients with an ANC count <1000 cells/μL. Degree of neutropenia was further stratified in the following 3 studies or 1024 out of the 1241 total procedures across all the 6 studies: 54.5% (N = 559) with ANC 500–1000 cells/μL; 32.8% (N = 336) with ANC <500 cells/μL; and 12.7% (N = 129) with ANC <200 cells/μL.[4,11,18] We excluded Kaw et al[16] from fever analysis because it is not clear if the 1 patient who developed a fever after undergoing a flexible sigmoidoscopy 3 days posttransplant was neutropenic or not. As detailed in this paper, this specific patient's thorough work-up for infectious source was negative, suggesting that the fever was not related to an infectious adverse event from endoscopy.

Procedures and Interventions

The number of each procedure type was not consistently indicated. Four studies provided data on procedural intervention. Among these studies, 59% (N = 705) of procedures involved endoscopic intervention as follows: 8.6% (N = 46) included hemostatic therapy and 77.1% (N = 544) included biopsy. Sphincterotomy and PEG tube placement were also performed, although their frequencies were not specified. Antibiotic use prior to endoscopy was noted in 5 out of the 6 studies, with 76.9% (N = 923) of procedures in these studies done after antibiotic administration.[4,10,11,16,18] Indications for procedures included nausea and/or vomiting, anemia, overt signs of luminal bleeding, dysphagia, odynophagia, and other. The frequencies of procedures done for each of these indications were mentioned in 2 studies.[4,11]

Endoscopic and Histologic Findings

Endoscopic and histologic findings across all studies included peptic ulcer, varices, other bleeding lesions, graft vs host disease, cytomegalovirus, candidiasis, *C difficile*, nonspecific inflammation (including gastritis and esophagitis), and other. The frequency of these findings was not consistently described.

Primary and Secondary Outcomes

Infection-related mortality within 30 days (duration of follow-up 3–30 days) was noted in 3 studies with pooled incidence of 0.0% (95% CI 0.0%–0.03%; I^2 = 0.0%) (Table, Figure 2).[4,10,18] Infectious adverse events within 7 days (duration of follow-up 2–7 days) were reported in 5 studies with pooled incidence of 7.3% (95% CI 0.00%–27.57%; I^2 = 98.6%) (Table, Figure 3).[4,10,11,17,18] Infectious adverse events include both documented new infection and new fever, and we also assessed these elements individually. New bacteremia was reported in all the 6 studies, and the pooled incidence was 2.4% (95% CI 0.03%–6.99%; I^2 = 87.3%) (Figure 4). New fever was noted in 5 studies, and the pooled incidence was 4.8% (95% CI 0.00%–9.74%; I^2 = 99%) (Figure 5).[4,10,11,17,18]

PEG tube placements were included in the study of Abu-Sbeih et al,[4] with 2 of 42 patients undergoing this procedure developing PEG site infection. PEG tube placements were not explicitly mentioned as an endoscopic intervention in the remaining studies and therefore not measured separately as an outcome.

Data on antibiotic use prior to endoscopy related to outcomes of infectious adverse events or bacteremia were available in 5 studies.[4,10,11,16,18] Kaw et al[16] had a total of 10 bone marrow transplant recipients undergoing EGD, flexible sigmoidoscopy, or colonoscopy. Seven of these patients were on antibiotics at the time of their procedure; no patients, including those without antibiotics, developed bacteremia. Vishny et al[18] included 36 bone marrow transplant recipients who were all receiving antibiotics and had an EGD, with no infectious adverse events noted. Abu-Sbeih et al[4] had 485 procedures in patients receiving antibiotics and the following 4 of those patients developed infectious adverse events within 1 week: 2 of these were

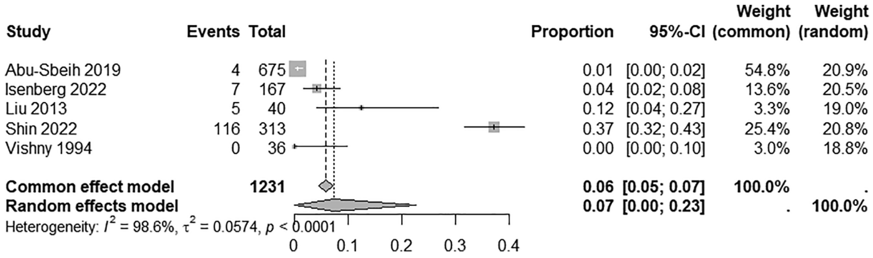

Figure 3. Forest plot of infectious adverse events.

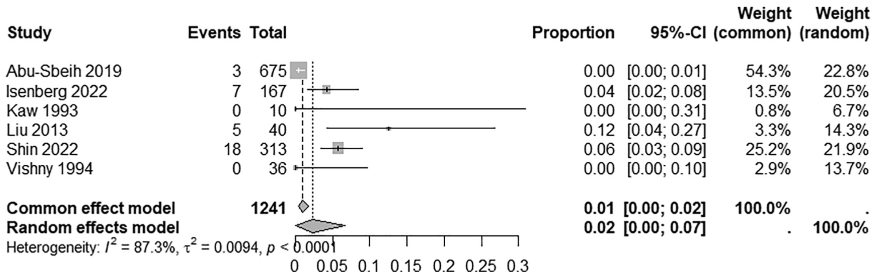

Figure 4. Forest plot of new bacteremia.

PEG tube site infections and 2 were fever of unknown origin (2 others developed gram-negative sepsis but had infections present at time of endoscopy). None of the 190 patients not receiving antibiotics in the study of Abu-Sbeih et al developed infectious adverse events within 1 week. Isenberg et al[10] reported rates of bacteremia to be similar in those receiving preprocedural antibiotics (4/94 (4.3%) and those not receiving antibiotics 3/73 (4.1%). The study of Shin et al[11] had 300 (96%) of 313 neutropenic patients who were receiving antibiotics at the time of the procedure; all infectious events occurred in those on antibiotics (116 [38.7%]). Most of these studies did not provide specific information on the indications for antibiotic use; only the study of Shin et al[11] mentioned indication, stating that "most" patients received antibiotics for prevention of neutropenic fever and not for the prevention of endoscopy-related adverse events, while some received antibiotics to treat an underlying medical condition. Random-effects meta-analysis of the 4 studies comparing infectious adverse events or bacteremia in those receiving antibiotics vs those not receiving antibiotics revealed odds ratio = 2.97; 95% CI 0.47–18.67; and $I^2 = 45\%$.[4,10,11,16]

Risk-of-Bias Assessment

All studies lacked a nonexposed cohort (neutropenic patients who did not undergo endoscopy) and therefore could only receive a maximum of 3 stars in the selection domain. (Supplementary Appendix D). For the selection domain, all studies received 3 stars. For the comparability domain, 2 studies received 1 star and 4 studies received 2 stars. For the outcome domain, 2 studies received 2 starts and 4 studies received 3 stars. Based on these findings, 5 out of the 6 studies were noted to have a low risk of bias (total score 7–9) and 1 study (Kaw et al) had a high risk of bias due to the total score being 6.

Discussion

This systematic review and meta-analysis suggests that the risk associated with endoscopy in neutropenic patients is relatively low. The pooled incidence for one of our coprimary end points, infection-related mortality was 0.0% within 30 days. The pooled incidence for our other coprimary end point, infectious adverse events within 7 days, was 7.3%, but this end point included outcomes of both bacteremia, which had a pooled incidence of 2.4%, and fever, with pooled incidence of 4.8%.

An important limitation of all studies included in our systematic review is the lack of a control group of neutropenic patients not undergoing endoscopy. In neutropenic patients in general, the risk of infection is largely dependent on the duration and degree of neutropenia. In 1 study, the risk of infection was 14% in those with ANC between 500 and 1000 and 24%–60% in those with ANC <1000.[19] Fever in neutropenic patients is estimated to occur in 10%–50% of patients with solid tumors and in >80% of those with hematologic malignancies.[20] Eighty-eight percent of procedures in our review were performed on patients with malignancy, the majority being hematologic. Therefore, the infection risk related to endoscopy appears lower than the infection risk seen overall in neutropenic patients.

The meta-analysis for infection-related mortality shows no evidence of statistical heterogeneity ($I^2 = 0$). In contrast, the meta-analysis of infectious adverse events shows dramatic heterogeneity with an I^2 of 98.6%. In meta-analyses with marked heterogeneity, it is important to assess the potential causes of heterogeneity and consider whether the summary statistic from the pooled studies should be accepted. Visual examination of the forest plot for infectious adverse events (Figure 3) shows that the study of Shin et al is an outlier and the major driver for the increased number

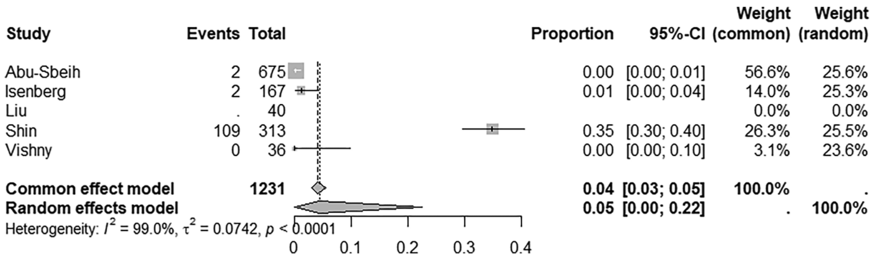

Figure 5. Forest plot of new fever.

of infectious adverse events in our review. While new fever occurred in 35 of 109 patients, only a smaller proportion of these patients developed bacteremia (N = 11; 10%), suggesting the reported infectious adverse events were not clinically consequential in many patients. Shin et al reported poor performance status and myelodysplastic syndrome as risk factors for infectious adverse events on multivariable analysis in patients on antibiotics, and these characteristics were present in 37% and 57% of their patients, respectively. Further study is needed to determine if certain subsets of neutropenic patients have higher risk.

Guidelines from the British Society of Gastroenterology in 2009 state that neutropenia predisposes to sepsis after endoscopy endoscopy and recommend antibiotic prophylaxis for patients with severe neutropenia (ANC <500 cells/μL),[2] and guidelines from the American Society for Gastrointestinal Endoscopy in 2015 state that patients with ANC <500 cells/μL and advanced hematologic malignancies are at increased risk for bacteremia and sepsis after GI endoscopy.[1] Both guidelines cite only 1 article to support these statements, a study from 1990 in which 9 of 47 bone marrow transplant patients undergoing EGD developed clinically evident bacteremia.[21] However, the population in this study was not patients with severe neutropenia—in fact, an inclusion criterion was ANC >500 cells/μL. Thus, these guideline statements regarding severe neutropenia were not based on data from patients with severe neutropenia. Since then, significantly more evidence has emerged. We found that 1155 (93%) of the cases included in our systematic review were from reports published after 2015, thereby enabling our review to provide far more evidence with more precise estimates of outcomes.

Societal guidelines suggest the use of prophylactic antibiotics when proceeding with endoscopy in patients with ANC <500 cells/μL again based on a single small retrospective study in patients who all had ANCs >500 cells/μL.[1,2,22] In our meta-analysis, the use of antibiotic prophylaxis prior to endoscopy was stated in 5 of the 6 studies.[4,10,11,16,18] Preprocedural antibiotic use was associated with a nonstatistically significant increase in infectious adverse events (odds ratio = 2.97; 95% CI 0.47–18.67), though this likely reflects confounding by indication, as antibiotics may have been preferentially administered to higher-risk patients. The observational nature of included studies and potential selection bias preclude definitive conclusions regarding antibiotic efficacy.

A 2007 abstract using the Nationwide Inpatient Sample database noted that among neutropenic patients with diagnostic indications such as GI bleeding, mortality was 49% less in those who underwent EGD and colonoscopy compared to those who did not.[23] Appropriate use of endoscopy may therefore in fact decrease in-hospital and all-cause mortality. Studies in pediatric patients with neutropenia and immunocompromised states has yielded results similar to those seen in the adult population.[6,7,24] In 1 study, both neutropenic and nonneutropenic pediatric patients underwent PEG tube placement with the same infection risk in both groups.[7] In another group of 148 pediatric patients with ANC <1000, none developed infectious adverse events following endoscopy.[24] And in a final study with 38 neutropenic pediatric cancer patients, there was only 1 infectious adverse event with fever and abdominal pain that resolved with 2 days of intravenous antibiotics.[6]

This study is not without limitations. Only 6 studies met inclusion criteria, demonstrating the limited data available and the need for additional large population-based studies. Out of these studies, 3 are clearly weighted more heavily than the others. The studies by Abu-Sbeih et al, Isenberg et al and Shin et al include 1155 procedures performed in neutropenic patients while the total number of procedures across the remaining 3 studies is only 86. Moreover, as mentioned above, no study had a comparator group. There is a known increased risk of infectious complications in neutropenic patients, regardless of whether or not they undergo endoscopy. Another limitation is the median short duration of follow-up for patients resulting in missed infectious adverse events. The follow-up period of infectious adverse events was only 1–7 days with a median follow-up of 5 days across all studies. It is conceivable that complications related to endoscopy may occur more than 1 week following endoscopy.

Further limitations include the lack of specific information on procedure type and procedural intervention. The available data did not allow us to assess if risk of infection varies with procedure type (eg, EGD, colonoscopy, PEG) or if infection increases with endoscopic intervention. In addition, immunosuppression is a known risk factor for infectious adverse events. While we know that 88% of patients included in our meta-analysis across all studies carried a diagnosis of malignancy and were neutropenic, other factors that may have led to further immunosuppression were not clear. The use of biologic or chemotherapeutic agents, steroids, or radiation therapy was not delineated. Finally, data were not available in the studies included in our systematic review to allow us to assess whether the risk varied with different severities or durations of neutropenia. Among the 1024 procedures were neutropenia was stratified, more than half had an ANC between 500 and 1000 cells/μL. This limits the generalizability of our findings to patients with severe neutropenia (ANC <500 cells/μL).

Conclusion

This systematic review and meta-analysis demonstrates that the risk of endoscopic procedures in neutropenic adults may be lower than was suggested in the past and fails to document a benefit of preprocedural antibiotics. These findings highlight the need for large population-based studies on the safety of endoscopy in neutropenia, and further suggest that neutropenia should not be a barrier to endoscopy when it is otherwise indicated.

Supplementary Materials

Material associated with this article can be found, in the online version, at https://doi:10.1016/j.gastha.2026.100994.

References

1. Committee ASoP, Khashab MA, Chithadi KV, et al. Antibiotic prophylaxis for GI endoscopy. Gastrointest Endosc 2015;81(1):81–89.
2. Allison MC, Sandoe JA, Tighe R, et al. Antibiotic prophylaxis in gastrointestinal endoscopy. Gut 2009; 58(6):869–880.
3. Freifeld AG, Bow EJ, Sepkowitz KA, et al. Clinical practice guideline for the use of antimicrobial agents in neutropenic patients with cancer: 2010 update by the Infectious Diseases Society of America. Clin Infect Dis 2011;52(4):e56–e93.
4. Abu-Sbeih H, Ali FS, Coronel E, et al. Safety of endoscopy in cancer patients with thrombocytopenia and neutropenia. Gastrointest Endosc 2019;89(5):937–949.e2.
5. Tong MC, Tadros M, Vaziri H. Endoscopy in neutropenic and/or thrombocytopenic patients. World J Gastroenterol 2015;21(46):13166–13176.
6. Buderus S, Sonderkotter H, Fleischhack G, et al. Diagnostic and therapeutic endoscopy in children and adolescents with cancer. Pediatr Hematol Oncol 2012;29(5):450–460.
7. Kaur S, Ceballos C, Bao R, et al. Percutaneous endoscopic gastrostomy tubes in pediatric bone marrow transplant patients. J Pediatr Gastroenterol Nutr 2013; 56(3):300–303.
8. Gorschluter M, Schmitz V, Mey U, et al. Endoscopy in patients with acute leukaemia after intensive chemotherapy. Leuk Res 2008;32(10):1510–1517.
9. Loganathan P, Mohan BP, Alderman D, et al. Safety of endoscopic procedures in thrombocytopenia and neutropenia: embracing assurance. Dig Dis Sci 2024; 69(7):2354–2362.
10. Isenberg Y, Zamstein N, Horesh N, et al. Risk factors for bacteremia after endoscopic procedures in hospitalized patients with a focus on neutropenia. J Clin Gastroenterol 2022;56(1):e58–e63.
11. Shin GY, Park JM, Lee DG, et al. Infectious events after endoscopic procedures in patients with neutropenia and hematologic diseases. Surg Endosc 2022;36(10):7360–7368.
12. Stroup DF, Berlin JA, Morton SC, et al. Meta-analysis of observational studies in epidemiology: a proposal for reporting. Meta-analysis of Observational Studies in Epidemiology (MOOSE) group. JAMA 2000;283(15):2008–2012.
13. Page MJ, Moher D, Bossuyt PM, et al. PRISMA 2020 explanation and elaboration: updated guidance and exemplars for reporting systematic reviews. BMJ 2021;372:n160.
14. McGowan J, Sampson M, Salzwedel DM, et al. PRESS peer review of electronic search strategies: 2015 guideline statement. J Clin Epidemiol 2016;75:40–46.
15. Lo CK, Mertz D, Loeb M. Newcastle-Ottawa Scale: comparing reviewers' to authors' assessments. BMC Med Res Methodol 2014;14:45.
16. Kaw M, Przepiorka D, Sekas G. Infectious complications of endoscopic procedures in bone marrow transplant recipients. Dig Dis Sci 1993;38(1):71–74.
17. Liu A, Meyer E, Johnston L, et al. Prevalence of graft versus host disease and cytomegalovirus infection in patients post-haematopoietic cell transplantation presenting with gastrointestinal symptoms. Aliment Pharmacol Ther 2013;38(8):955–966.
18. Vishny ML, Blades EW, Creger RJ, et al. Role of upper endoscopy in evaluation of upper gastrointestinal symptoms in patients undergoing bone marrow transplantation. Cancer Invest 1994;12(4):384–389.
19. Bodey GP, Buckley M, Sathe YS, et al. Quantitative relationships between circulating leukocytes and infection in patients with acute leukemia. Ann Intern Med 1966; 64(2):328–340.
20. Klastersky J. Management of fever in neutropenic patients with different risks of complications. Clin Infect Dis 2004;39 Suppl 1:S32–S37.
21. Bianco JA, Pepe MS, Higano C, et al. Prevalence of clinically relevant bacteremia after upper gastrointestinal endoscopy in bone marrow transplant recipients. Am J Med 1990;89(2):134–136.
22. Rey JR, Axon A, Budzynska A, et al. Guidelines of the European Society of Gastrointestinal Endoscopy (E.S.G. E.) antibiotic prophylaxis for gastrointestinal endoscopy. European Society of Gastrointestinal Endoscopy. Endoscopy 1998;30(3):318–324.
23. Rahimi E, Batra S, Siddiqui S, Guha S. Risks of endoscopic complications in patients with neutropenia: a population-based U.S. study: 2245. Am J Gastroenterol 2014;109:S652.
24. Khan K, Schwarzenberg SJ, Sharp H, et al. Diagnostic endoscopy in children after hematopoietic stem cell transplantation. Gastrointest Endosc 2006;64(3):379–385, quiz 389-385.

Received June 14, 2025. Accepted May 1, 2026.

Correspondence:
Address correspondence to: Janak Bahirwani, MD, Kadlec Regional Medical Center, 1270 Lee Blvd, Richland, Washington 99352. e-mail: Janak.bahirwani@kadlec.org or Janak.bahirwani@wsu.edu.

Authors' Contributions:
Janak Bahirwani: Data extraction, analysis and interpretation of data, and drafting and revising the manuscript. Rishika Chugh: Conception and design, analysis and interpretation of data, data extraction, drafting and revising article, and final approval of the manuscript. Ashley N. Tran: Data extraction, analysis and interpretation of data, critical revision of the article for important intellectual content, and final approval of the manuscript. Amy Ogurick: Data extraction, critical revision of the article for important intellectual content, and final approval of the manuscript. Alyssa A. Grimshaw: Database search, critical revision of the article for important intellectual content, and final approval of the manuscript. Maria Ciarleglio: Analysis and interpretation of data, critical revision of the article for important intellectual content, and final approval of the manuscript. Yanhong Deng: Analysis and interpretation of data, critical revision of the article for important intellectual content, and final approval of the manuscript. Badr Al-Bawardy: Conception and design, critical revision of the article for important intellectual content, and final approval of the manuscript. Kenneth W. Hung: Conception and design, critical revision of the article for important intellectual content, and final approval of the manuscript. Loren Laine: Conception and design, analysis and interpretation of data, critical revision of the article for important intellectual content, and final approval of the manuscript.

Conflicts of Interest:
The author discloses the following: Rishika Chugh: Academic advisory board Bristol Myers Squibb. The remaining authors disclose no conflicts.

Funding:
The authors report no funding.

Ethical Statement:
Institutional review board approval was not required, as this study involved analysis of publicly available data and did not include any identifiable patient information.

Data Transparency Statement:
All data analyzed in this study are derived from previously published articles, which are cited within the manuscript. No new data were generated. Additional details regarding the dataset and analytic methods are available from the corresponding author upon reasonable request.

Reporting Guidelines:
PRISMA.

Gastro Hep Advances, Volume 5

Supplemental information

Mortality and Infectious Adverse Events in Neutropenic Patients Undergoing Gastrointestinal Endoscopic Procedures: A Systematic Review and Meta-Analysis

Janak Bahirwani, Rishika Chugh, Ashley N. Tran, Amy Ogurick, Alyssa A. Grimshaw, Maria Ciarleglio, Yanhong Deng, Badr Al-Bawardy, Kenneth W. Hung, and Loren Laine

Supplementary File

Title: Mortality and Infectious Adverse Events in Neutropenic Patients undergoing Gastrointestinal Endoscopic Procedures: A Systematic Review and Meta-Analysis

Authors: Janak Bahirwani, MD[1,8] Rishika Chugh MD[2], Ashley N. Tran MD[3], Amy Ogurick MD[4], Alyssa A. Grimshaw MSLIS, MPH[5], Maria Ciarleglio PhD[4], Yanhong Deng MPH[4], Badr Al-Bawardy MD4,6, Kenneth W. Hung MD, MS[4], Loren Laine MD[4,7]

1. Kadlec Regional Medical Center, Richland, Washington, USA
2. University of California, San Francisco, San Francisco, California, USA
3. St Luke's University Health Network, Bethlehem, Pennsylvania, USA
4. Yale School of Medicine, New Haven, Connecticut, USA
5. Harvey Cushing/John Hay Whitney Medical Library, Yale University, New Haven, Connecticut, USA
6. King Faisal Specialist Hospital, Riyadh, Saudi Arabia
7. VA Connecticut Health Care System, West Haven, Connecticut, USA
8. Elson S. Floyd College of Medicine at Washington State University Tri-cities campus

Appendix A: Reporting Guideline Checklists

PRISMA 2020 Main Checklist

Topic	No.	Item	Location where item is reported
**TITLE**			
**Title**	1	Identify the report as a systematic review.	LN1-2
**ABSTRACT**			
**Abstract**	2	See the PRISMA 2020 for Abstracts checklist	Appendix 1
**INTRODUCTION**			
**Rationale**	3	Describe the rationale for the review in the context of existing knowledge.	LN140-169
**Objectives**	4	Provide an explicit statement of the objective(s) or question(s) the review addresses.	LN1171-173
**METHODS**			
**Eligibility criteria**	5	Specify the inclusion and exclusion criteria for the review and how studies were grouped for the syntheses.	LN198-203
**Information sources**	6	Specify all databases, registers, websites, organisations, reference lists and other sources searched or consulted to identify studies. Specify the date when each source was last searched or consulted.	LN182-187
**Search strategy**	7	Present the full search strategies for all databases, registers and websites, including any filters and limits used.	Appendix B
**Selection process**	8	Specify the methods used to decide whether a study met the inclusion criteria of the review, including how many reviewers screened each record and each report retrieved, whether they worked independently, and if applicable, details of automation tools used in the process.	LN189-192
**Data collection process**	9	Specify the methods used to collect data from reports, including how many reviewers collected data from each report, whether they worked independently, any processes for obtaining or confirming data from study investigators, and if applicable, details of automation tools used in the process.	LN206-208
**Data items**	10a	List and define all outcomes for which data were sought. Specify whether all results that were compatible with each outcome domain in each study were sought (e.g. for all measures, time points, analyses), and if not, the methods used to decide which results to collect.	LN218-240
	10b	List and define all other variables for which data were sought (e.g. participant and intervention characteristics, funding sources). Describe any assumptions made about any missing or unclear information.	LN234-240
**Study risk of bias assessment**	11	Specify the methods used to assess risk of bias in the included studies, including details of the tool(s) used, how many reviewers assessed each study and whether they worked independently, and if applicable, details of automation tools used in the process.	LN209-215

Topic	No.	Item	Location where item is reported
**Effect measures**	12	Specify for each outcome the effect measure(s) (e.g. risk ratio, mean difference) used in the synthesis or presentation of results.	LN243-250
**Synthesis methods**	13a	Describe the processes used to decide which studies were eligible for each synthesis (e.g. tabulating the study intervention characteristics and comparing against the planned groups for each synthesis (item 5)).	LN243-250
	13b	Describe any methods required to prepare the data for presentation or synthesis, such as handling of missing summary statistics, or data conversions.	LN218-250
	13c	Describe any methods used to tabulate or visually display results of individual studies and syntheses.	LN218-250
	13d	Describe any methods used to synthesize results and provide a rationale for the choice(s). If meta-analysis was performed, describe the model(s), method(s) to identify the presence and extent of statistical heterogeneity, and software package(s) used.	LN218-250
	13e	Describe any methods used to explore possible causes of heterogeneity among study results (e.g. subgroup analysis, meta-regression).	LN252-256
	13f	Describe any sensitivity analyses conducted to assess robustness of the synthesized results.	LN252-256
**Reporting bias assessment**	14	Describe any methods used to assess risk of bias due to missing results in a synthesis (arising from reporting biases).	LN209-215
**Certainty assessment**	15	Describe any methods used to assess certainty (or confidence) in the body of evidence for an outcome.	LN252-256
**RESULTS**			
**Study selection**	16a	Describe the results of the search and selection process, from the number of records identified in the search to the number of studies included in the review, ideally using a flow diagram.	LN259-263
	16b	Cite studies that might appear to meet the inclusion criteria, but which were excluded, and explain why they were excluded.	Appendix C
**Study characteristics**	17	Cite each included study and present its characteristics.	LN263-286
**Risk of bias in studies**	18	Present assessments of risk of bias for each included study.	LN329-335
**Results of individual studies**	19	For all outcomes, present, for each study: (a) summary statistics for each group (where appropriate) and (b) an effect estimate and its precision (e.g. confidence/credible interval), ideally using structured tables or plots.	LN263-335
**Results of syntheses**	20a	For each synthesis, briefly summarise the characteristics and risk of bias among contributing studies.	LN263-335

Topic	No.	Item	Location where item is reported
	20b	Present results of all statistical syntheses conducted. If meta-analysis was done, present for each the summary estimate and its precision (e.g. confidence/credible interval) and measures of statistical heterogeneity. If comparing groups, describe the direction of the effect.	LN307-335
	20c	Present results of all investigations of possible causes of heterogeneity among study results.	LN307-335
	20d	Present results of all sensitivity analyses conducted to assess the robustness of the synthesized results.	LN307-335
**Reporting biases**	21	Present assessments of risk of bias due to missing results (arising from reporting biases) for each synthesis assessed.	LN338-344
**Certainty of evidence**	22	Present assessments of certainty (or confidence) in the body of evidence for each outcome assessed.	LN307-344
**DISCUSSION**			
**Discussion**	23a	Provide a general interpretation of the results in the context of other evidence.	LN347-404
	23b	Discuss any limitations of the evidence included in the review.	LN407-417
	23c	Discuss any limitations of the review processes used.	LN419-426
	23d	Discuss implications of the results for practice, policy, and future research.	LN428-432
**OTHER INFORMATION**			
**Registration and protocol**	24a	Provide registration information for the review, including register name and registration number, or state that the review was not registered.	LN176-179
	24b	Indicate where the review protocol can be accessed, or state that a protocol was not prepared.	LN176-179
	24c	Describe and explain any amendments to information provided at registration or in the protocol.	Available on PROSPERO
**Support**	25	Describe sources of financial or non-financial support for the review, and the role of the funders or sponsors in the review.	LN30
**Competing interests**	26	Declare any competing interests of review authors.	LN32-44
**Availability of data, code and other materials**	27	Report which of the following are publicly available and where they can be found: template data collection forms; data extracted from included studies; data used for all analyses; analytic code; any other materials used in the review.	Upon Request

From: Page MJ, McKenzie JE, Bossuyt PM, Boutron I, Hoffmann TC, Mulrow CD, et al. The PRISMA 2020 statement: an updated guideline for reporting systematic reviews. MetaArXiv. 2020, September 14. DOI: 10.31222/osf.io/v7gm2. For more information, visit: www.prisma-statement.org

PRISMA Abstract Checklist

Topic	No.	Item	Reported?
**TITLE**			
**Title**	1	Identify the report as a systematic review.	Yes
**BACKGROUND**			
**Objectives**	2	Provide an explicit statement of the main objective(s) or question(s) the review addresses.	Yes
**METHODS**			
**Eligibility criteria**	3	Specify the inclusion and exclusion criteria for the review.	Yes
**Information sources**	4	Specify the information sources (e.g. databases, registers) used to identify studies and the date when each was last searched.	Yes
**Risk of bias**	5	Specify the methods used to assess risk of bias in the included studies.	Yes
**Synthesis of results**	6	Specify the methods used to present and synthesize results.	Yes
**RESULTS**			
**Included studies**	7	Give the total number of included studies and participants and summarise relevant characteristics of studies.	Yes
**Synthesis of results**	8	Present results for main outcomes, preferably indicating the number of included studies and participants for each. If meta-analysis was done, report the summary estimate and confidence/credible interval. If comparing groups, indicate the direction of the effect (i.e. which group is favoured).	Yes
**DISCUSSION**			
**Limitations of evidence**	9	Provide a brief summary of the limitations of the evidence included in the review (e.g. study risk of bias, inconsistency and imprecision).	Yes
**Interpretation**	10	Provide a general interpretation of the results and important implications.	Yes
**OTHER**			
**Funding**	11	Specify the primary source of funding for the review.	Yes
**Registration**	12	Provide the register name and registration number.	Yes

MOOSE Checklist for Meta-analyses of Observational Studies

Item No	Recommendation	Reported on Page No
Reporting of background should include		
1	Problem definition	6-7
2	Hypothesis statement	6-7
3	Description of study outcome(s)	6-7
4	Type of exposure or intervention used	6-7
5	Type of study designs used	6-7
6	Study population	6-7
Reporting of search strategy should include		
7	Qualifications of searchers (eg, librarians and investigators)	7, Title page
8	Search strategy, including time period included in the synthesis and key words	7, Appendix B
9	Effort to include all available studies, including contact with authors	7-8
10	Databases and registries searched	7
11	Search software used, name and version, including special features used (eg, explosion)	7
12	Use of hand searching (eg, reference lists of obtained articles)	8
13	List of citations located and those excluded, including justification	Figure 1
14	Method of addressing articles published in languages other than English	7-8
15	Method of handling abstracts and unpublished studies	7-8
16	Description of any contact with authors	7-8
Reporting of methods should include		
17	Description of relevance or appropriateness of studies assembled for assessing the hypothesis to be tested	8-10
18	Rationale for the selection and coding of data (eg, sound clinical principles or convenience)	8-10
19	Documentation of how data were classified and coded (eg, multiple raters, blinding and interrater reliability)	8-10
20	Assessment of confounding (eg, comparability of cases and controls in studies where appropriate)	8-10
21	Assessment of study quality, including blinding of quality assessors, stratification or regression on possible predictors of study results	8-10
22	Assessment of heterogeneity	8-10
23	Description of statistical methods (eg, complete description of fixed or random effects models, justification of whether the chosen models account for predictors of study results, dose-response models, or cumulative meta-analysis) in sufficient detail to be replicated	8-10

24	Provision of appropriate tables and graphics	Table 1<br>Figure 2-5
Reporting of results should include		
25	Graphic summarizing individual study estimates and overall estimate	Table 1<br>Figure 2-5
26	Table giving descriptive information for each study included	Table 1
27	Results of sensitivity testing (eg, subgroup analysis)	Figure 2-5
28	Indication of statistical uncertainty of findings	10-16

Item No	Recommendation	Reported on Page No
Reporting of discussion should include		
29	Quantitative assessment of bias (eg, publication bias)	13
30	Justification for exclusion (eg, exclusion of non-English language citations)	16-17
31	Assessment of quality of included studies	13-15
Reporting of conclusions should include		
32	Consideration of alternative explanations for observed results	17
33	Generalization of the conclusions (ie, appropriate for the data presented and within the domain of the literature review)	17
34	Guidelines for future research	17
35	Disclosure of funding source	1

From: Stroup DF, Berlin JA, Morton SC, et al, for the Meta-analysis Of Observational Studies in Epidemiology (MOOSE) Group. Meta-analysis of Observational Studies in Epidemiology. A Proposal for Reporting. *JAMA*. 2000;283(15):2008-2012. doi: 10.1001/jama.283.15.2008.

Appendix B: Search Strategies

Ovid Embase

1 exp digestive tract endoscopy/
2 (endoscop* adj3 (digestive or gastrointestinal)).tw,kw.
3 (balloon enteroscop* or (push and pull endoscop*) or double balloon endoscop* or single balloon endoscop* or Colonoscop* or Sigmoidoscop* or Proctosigmoidoscop* or Duodenoscop*).tw,kw.
4 (Esophagoscop* or Gastroscop* or Proctoscop* or strip biosp* or esophagogastroduodenoscop* or endoscopic ultrasound* or percutaneous endoscopic gastrostom*).tw,kw.
5 (Endoscopic adj (Mucosal or submucosal) adj3 (Resection* or Dissection*)).tw,kw.
6 (endoscop* adj3 retrograd* adj3 cholangio*).tw,kw.
7 (EGD or ERCP or EUS or PEG).ti,ab.
8 1 or 2 or 3 or 4 or 5 or 6 or 7
9 exp Leukopenia/
10 (neutropaeni* or neutropeni*).tw,kw.
11 (neutrophil* adj3 (dysfunction* or disease* or disorder*)).tw,kw.
12 (leukopeni* or leukocytopeni* or agranulocytoses or agranulocytosis or granulocytopeni* or lymphopeni* or lymphocytopeni*).tw,kw.
13 (pancytopeni* or cytopeni* or bicytopeni*).tw,kw.
14 9 or 10 or 11 or 12 or 13
15 8 and 14
16 exp animal/
17 exp animal/ and exp human/
18 16 not 17
19 15 not 18
20 exp embryo/ or exp infant/ or exp juvenile/
21 (exp embryo/ or exp infant/ or exp juvenile/) and exp adult/
22 20 not 21
23 19 not 22
24 limit 23 to conference abstracts
25 23 not 24
26 limit 25 to english language

Ovid MEDLINE(R) ALL

1 exp Endoscopy, Gastrointestinal/
2 (endoscop* adj3 (digestive or gastrointestinal)).tw,kf.

3 (balloon enteroscop* or (push and pull endoscop*) or double balloon endoscop* or single balloon endoscop* or Colonoscop* or Sigmoidoscop* or Proctosigmoidoscop* or Duodenoscop*).tw,kf.
4 (Esophagoscop* or Gastroscop* or Proctoscop* or strip biosp* or esophagogastroduodenoscop* or endoscopic ultrasound* or percutaneous endoscopic gastrostom*).tw,kf.
5 (Endoscopic adj (Mucosal or submucosal) adj3 (Resection* or Dissection*)).tw,kf.
6 (endoscop* adj3 retrograd* adj3 cholangio*).tw,kf.
7 (EGD or ERCP or EUS or PEG).ti,ab.
8 or/1-7
9 exp Leukopenia/
10 (neutropaeni* or neutropeni*).tw,kf.
11 (neutrophil* adj3 (dysfunction* or disease* or disorder*)).tw,kf.
12 (leukopeni* or leukocytopeni* or agranulocytoses or agranulocytosis or granulocytopeni* or lymphopeni* or lymphocytopeni*).tw,kf.
13 (pancytopeni* or cytopeni* or bicytopeni*).tw,kf.
14 or/9-13
15 8 and 14
16 exp animal/
17 exp animal/ and exp human/
18 16 not 17
19 15 not 18
20 exp child/ or exp infant/
21 (exp child/ or exp infant/) and exp adults/
22 20 not 21
23 19 not 22
24 limit 23 to english language

Web of Science

#1 TS=(endoscop* near/3 (digestive or gastrointestinal)) or TS=("balloon enteroscop*" or "push and pull endoscop*" or "double balloon endoscop*" or "single balloon endoscop*" or Colonoscop* or Sigmoidoscop* or Proctosigmoidoscop* or Duodenoscop*) or TS=(Esophagoscop* or Gastroscop* or Proctoscop* or "strip biosp*" or sophagogastroduodenoscop* or "endoscopic ultrasound*" or "percutaneous endoscopic gastrostom*") or TS=(Endoscopic near/1 (Mucosal or submucosal) near/3 (Resection* or Dissection*)) or TS=(endoscop* near/3 retrograd* near/3 cholangio*) or TS=(EGD or ERCP or EUS or PEG)
#2 TS=(neutropaeni* or neutropeni*) or TS=(neutrophil* near/3 (dysfunction* or disease* or disorder*)) or TS=(leukopeni* or leukocytopeni* or agranulocytoses or agranulocytosis or granulocytopeni* or lymphopeni* or lymphocytopeni*) or TS=(pancytopeni* or cytopeni* or bicytopeni*)
#3 #1 and #2

Scopus

(TITLE-ABS-KEY (neutropaeni* OR neutropeni*) OR TITLE-ABS-KEY (neutrophil* W/3 (dysfunction* OR disease* OR disorder*)) OR TITLE-ABS-KEY (leukopeni* OR leukocytopeni* OR agranulocytoses OR agranulocytosis OR granulocytopeni* OR lymphopeni* OR lymphocytopeni*) OR TITLE-ABS-KEY (pancytopeni* OR cytopeni* OR bicytopeni*)) AND (TITLE-ABS-KEY (endoscop* W/3 (digestive OR gastrointestinal)) OR TITLE-ABS-KEY ("balloon enteroscop*" OR "push and pull endoscop*" OR "double balloon endoscop*" OR "single balloon endoscop*" OR colonoscop* OR sigmoidoscop* OR proctosigmoidoscop* OR duodenoscop*) OR TITLE-ABS-KEY (esophagoscop* OR gastroscop* OR proctoscop* OR "strip biosp*" OR sophagogastroduodenoscop* OR "endoscopic ultrasound*" OR "percutaneous endoscopic gastrostom*") OR TITLE-ABS-KEY (endoscopic W/1 (mucosal OR submucosal) W/3 (resection* OR dissection*)) OR TITLE-ABS-KEY (endoscop* W/3 retrograd* W/3 cholangio*) OR TITLE-ABS-KEY (egd OR ercp OR eus OR peg)) AND (LIMIT-TO (LANGUAGE , "English"))

Cochrane Library

#1 (endoscop* near/3 (digestive or gastrointestinal)):ti,ab or ("balloon enteroscop*" or "push and pull endoscop*" or "double balloon endoscop*" or "single balloon endoscop*" or Colonoscop* or Sigmoidoscop* or Proctosigmoidoscop* or Duodenoscop*):ti,ab or (Esophagoscop* or Gastroscop* or Proctoscop* or "strip biosp*" or sophagogastroduodenoscop* or "endoscopic ultrasound*" or "percutaneous endoscopic gastrostom*"):ti,ab or (Endoscopic near/1 (Mucosal or submucosal) near/3 (Resection* or Dissection*)):ti,ab or (endoscop* near/3 retrograd* near/3 cholangio*):ti,ab or (EGD or ERCP or EUS or PEG):ti,ab
#2 (neutropaeni* or neutropeni*):ti,ab or (neutrophil* near/3 (dysfunction* or disease* or disorder*)):ti,ab or (leukopeni* or leukocytopeni* or agranulocytoses or agranulocytosis or granulocytopeni* or lymphopeni* or lymphocytopeni*):ti,ab or (pancytopeni* or cytopeni* or bicytopeni*):ti,ab
#3 #1 and #2

PubMed

(neutropaeni* or neutropeni* or neutrophil* dysfunction* or neutrophil* disease* or neutrophil* disorder* or leukopeni* or leukocytopeni* or agranulocytoses or agranulocytosis or granulocytopeni* or lymphopeni* or lymphocytopeni* or pancytopeni* or cytopeni* or bicytopeni*) AND ((digestive endoscop*[Title/Abstract] OR gastrointestinal endoscop*[Title/Abstract] OR "balloon enteroscop*"[Title/Abstract] OR "push and pull endoscop*"[Title/Abstract] OR "double balloon endoscop*"[Title/Abstract] OR "single balloon endoscop*"[Title/Abstract] OR Colonoscop*[Title/Abstract] OR Sigmoidoscop*[Title/Abstract] OR Proctosigmoidoscop*[Title/Abstract] OR Duodenoscop*[Title/Abstract] OR Esophagoscop*[Title/Abstract] OR Gastroscop*[Title/Abstract] OR Proctoscop*[Title/Abstract] OR "strip biosp*"[Title/Abstract] OR

sophagogastroduodenoscop*[Title/Abstract] OR "endoscopic ultrasound*"[Title/Abstract] OR "percutaneous endoscopic gastrostom*"[Title/Abstract] OR endoscopic mucosal resection*[Title/Abstract] OR endoscopic submucosal resection*[Title/Abstract] OR endoscopic submucosal dissection*[Title/Abstract] OR endoscopic mucosal dissection*[Title/Abstract] OR endoscop* retrograd* cholangio*[Title/Abstract] OR EGD[Title/Abstract] OR ERCP[Title/Abstract] OR EUS[Title/Abstract] OR PEG[Title/Abstract]))

Google Scholar

neutropeni* gastrointestinal endoscopy

Appendix C: Excluded Studies Table

First Author Last Name	Year	Title	Journal	Exclusion Reason
Abu-Sbeih	2018	Mo1107 PREDICTORS OF OVERALL SURVIVAL IN CANCER PATIENTS WHO HAD ENDOSCOPIC EVALUATION IN THE SETTING OF NEUTROPENIA AND THROMBOCYTOPENIA	Gastrointestinal Endoscopy	Not enough details
Abu-Sbeih	2019	Neutropenic Colitis - Clinical Features, Treatment and Outcomes	Gastroenterology	Wrong outcomes
Agarwal	2016	Sepsis in a case of severe neutropenia unresponsive to G-CSF; a rare sequelae of chronic hepatitis C	American Journal of Gastroenterology	Wrong study design
AlAshgar	2009	Defecation of a "colon cast" as a rare presentation of acute graft-versus-host disease	Annals of Saudi Medicine	Wrong study design
Al-Azzawi	2016	ERCP for septic cholangitis through a peg tube, a life-saving procedure using an alternative access route to approach the biliary tree when the regular ERCP fails	American Journal of Gastroenterology	Wrong study design
Allen	2013	Efficacy and safety of treatment of hepatitis c in patients with inflammatory bowel disease	Clinical Gastroenterology and Hepatology	Wrong outcomes
Apanasenko	2018	A case of cytomegalovirus-induced pancytopenia with CMV colitis	HemaSphere	Wrong study design
Arai	1990	[Cyclic neutropenia complicated of non-Hodgkin lymphoma]	Rinsho Ketsueki - Japanese Journal of Clinical Hematology	Wrong study design
Armstrong	2018	Protracted severe systemic cytomegalovirus disease in an immunosuppressed patient with ulcerative colitis	Frontline Gastroenterology	Wrong study design
Auguste	1986	Postchemotherapy esophagitis: The endoscopic diagnosis and its impact on survival	Journal of Surgical Oncology	Wrong outcomes
Banerjee	2017	Role of emergency endoscopic decompression of colon in a case of compartment like syndrome of left lower limb secondary to severe acute pancreatitis	Surgical Endoscopy and Other Interventional Techniques	Wrong study design

Bernal	2016	Recurrent right-sided pleural effusion secondary to a pancreatic-pleural fistula: An endoscopic solution to a rare complication	American Journal of Gastroenterology	Wrong study design
Bernal	2017	Recurrent pleural effusion secondary to a pancreatic-pleural fistula treated endoscopically	American Journal of Case Reports	Wrong study design
Bitton	2016	Cytomegalovirus (CMV) colitis trigerring inflammatory bowel disease (IBD) in an immunocompetent adult: A case report and review of the literature	Canadian Journal of Gastroenterology and Hepatology. Conference	Wrong study design
Bolon	2017	Hemophagocytic lymphohistiocytosis (HLH), a rare and catastrophic cause of GI Bleeding	South African Gastroenterology Review	Wrong study design
Candiani	2019	Black esophagus: Acute esophageal necrosis syndrome: Case report	Italian Journal of Medicine	Wrong study design
Carroccio	2003	Autoimmune enteropathy and colitis in an adult patient	Digestive Diseases and Sciences	Wrong study design
Chen	2019	Additional Radiotherapy for Superficial Esophageal Cancer Following Endoscopic Submucosal Dissection (ESD): A Single Center Retrospective Study	Nanoscience and Nanotechnology Letters	Wrong intervention
Chisti	2013	Dasatinib-induced haemorrhagic colitis in chronic myeloid leukaemia (CML) in blast crisis	BMJ Case Reports.	Wrong study design
Choi	2011	A case of Crohn's disease with improvement after azathioprine-induced pancytopenia	Case Reports in Gastroenterology	Wrong study design
Chong	2005	Human immunodeficiency virus and endoscopy: Experience of a general hospital in Singapore	Journal of Gastroenterology and Hepatology (Australia)	Wrong patient population
Cortes-Flores	2015	Long-term outcome after percutaneous endoscopic gastrostomy in geriatric Mexican patients	Geriatrics and Gerontology International	Wrong patient population
Crudeli	2017	Infection following lower gastrointestinal endoscopy: silent risk or non-event?	Endoscopy	Wrong study design
Docker	2010	[Gastric neuroendocrine tumors in a woman with systemic lupus erythematosus]	Deutsche Medizinische Wochenschrift	Wrong study design
Dworkin	1992	The safety and efficacy of gi endoscopy in patients with acute-leukemia - a review of 27 cases	International Journal of Oncology	Wrong outcomes

Elden	2016	A case of inflammatory bowel disease and a rare lymphoma on azathioprine therapy	American Journal of Gastroenterology	Wrong study design
Elfeki	2014	Abatacept use in graft-versus-host disease after orthotopic liver transplantation: A case report	Transplantation Proceedings	Wrong study design
ElRafei	2018	Splenomegaly with a side of sinister portal hypertension	American Journal of Tropical Medicine and Hygiene	Wrong study design
Endoscopy	1999	Infection Control During Gastrointestinal Endoscopy	Gastrointestinal Endoscopy	Wrong study design
Forbes	1995	A prospective study of screening upper gastrointestinal (GI) endoscopy prior to and after bone marrow transplantation (BMT)	Aust N Z J Med	Not enough details
Gorschluter	2008	Endoscopy in patients with acute leukaemia after intensive chemotherapy	Leukemia Research	Wrong patient population
Greene	1974	Esophagoscopy as a source of Pseudomonas aeruginosa sepsis in patients with acute leukemia: the need for sterilization of endoscopes	Gastroenterology	Wrong study design
Han	2019	The Risk of Infectious Adverse Events after Eus-Guided Fine Needle Biopsy	Gastrointestinal Endoscopy	Wrong patient population
Han	2019	Mo1060 THE RISK OF INFECTIOUS ADVERSE EVENTS AFTER EUS-GUIDED FINE NEEDLE BIOPSY	Gastrointestinal	Not enough details
Hefazi	2016	Safety and efficacy of fecal microbiota transplantation for recurrent clostridium infection in patients with hematologic malignancies	Blood	Wrong patient population
Hermes	2011	[Non-small-cell carcinoma of the lung with invasive Aspergillus infection after chemotherapy]	Deutsche Medizinische Wochenschrift	Non-English language
Hess	1997	Botryomycosis causing giant ulcers of the esophagus: A case report	Gastrointestinal Endoscopy	Wrong study design
Jafri	2009	Utility and safety of endoscopic procedures in neutropenic patients with gastrointestinal bleeding	Gastrointestinal Endoscopy	Not enough details
Janssen	2004	Frequency of bacteremia after linear EUS of the upper GI tract with and without FNA	Gastrointestinal Endoscopy	Wrong patient population

Jiang	2012	Endoscopic stenting and concurrent chemoradiotherapy for advanced esophageal cancer: a case-control study	World Journal of Gastroenterology	Wrong patient population
Kara	2016	Survival After Percutaneous Endoscopic Gastrostomy in Older Adults With Neurologic Disorders	Nutrition in Clinical Practice	Wrong patient population
Khan	2004	Emerging bacterial resistance patterns in febrile neutropenic patients: experience at a tertiary care hospital in Pakistan	Journal of Pakistan	Wrong patient population
Leharova	2019	Incidence of blood stream infection (BSI) associated with endoscopic retrograde cholangiopancreatography in a tertiary hospital; 3 years prospective surveillance study	Antimicrobial Resistance and Infection Control.	Wrong patient population
Lorenz	1996	[Antibiotic prophylaxis using cefuroxime in bile duct endoscopy]	Deutsche Medizinische Wochenschrift	Non-English language
Nagata	2011	Diagnostic value of antigenemia assay for cytomegalovirus gastrointestinal disease in immunocompromised patients	World Journal of Gastroenterology	Wrong outcomes
Nevah	2014	Transnasal PEG tube placement in patients with head and neck cancer	Gastrointestinal Endoscopy	Not enough details
Otaki	2014	Sa1488 Retrospective Review of 100 Endoscopic Evaluations of Patients on Bone Marrow Transplant Service for GVHD	Gastrointestinal Endoscopy	Not enough details
Perez	2002	Endoscopy in patients receiving radiation therapy to the thorax	Digestive Diseases and Sciences	Wrong patient population
Rajan	2020	Tu1078 DOES THROMBOCYTOPENIA PREDICT POOR OUTCOME AFTER ENDOSCOPY FOR EVALUATION OF GRAFT-VERSUS-HOST DISEASE IN â€¦	Gastrointestinal Endoscopy	Not enough details
Ross	2013	Endoscopy in Hematologic Malignancies	Gastrointestinal Endoscopy in the Cancer Patient	Wrong study design
Soylu	2005	Overt gastrointestinal bleeding in haematologic neoplasms	Digestive and Liver Disease	Wrong outcomes

Tong	2014	Endoscopy in Neutropenic and/or Thrombocytopenic Patients: Review of Current Evidence and Development of Clinical Recommendations: 1842	Official journal of the American College of Gastroenterology | ACG	Wrong study design
Tong	2015	Endoscopy in neutropenic and/or thrombocytopenic patients	World Journal of Gastroenterology	Wrong study design
Tsirigotis	2008	Keratinocyte growth factor is effective in the prevention of intestinal mucositis in patients with hematological malignancies treated with high-dose chemotherapy and autologous hematopoietic SCT: A video-capsule endoscopy study	Bone Marrow Transplantation	Wrong patient population
Wheeler	1987	Esophagitis in the immunocompromised host: role of esophagoscopy in diagnosis	Reviews of Infectious Diseases	Not enough details
Yamazaki	2016	Impact of prophylactic percutaneous endoscopic gastrostomy tube placement on treatment tolerance in head and neck cancer patients treated with cetuximab plus radiation	Japanese Journal of Clinical Oncology	Wrong outcomes
Ye	2017	Therapeutic ERCP safely performed in patients with leukemia: A single center experience	Journal of Digestive Diseases	Not enough details

Appendix D: Risk of bias

Study	Selection (0-3)	Comparability (0-2)	Outcomes (0-3)	Total score	Overall risk of bias
Abu-Sbeih et al.	4	2	3	9	Low
Isenberg et al.	4	2	3	9	Low
Kaw et al.	3	1	2	6	High
Liu et al.	3	1	3	7	Low
Shin et al.	4	2	3	9	Low
Vishny et al.	4	2	2	8	Low
